# Effective eradication of acute myeloid leukemia stem cells with FLT3-directed antibody-drug conjugates

**DOI:** 10.1038/s41375-024-02510-5

**Published:** 2025-01-27

**Authors:** Marina Able, Marc-André Kasper, Binje Vick, Jonathan Schwach, Xiang Gao, Saskia Schmitt, Belay Tizazu, Amrei Fischer, Sarah Künzl, Marit Leilich, Isabelle Mai, Philipp Ochtrop, Andreas Stengl, Mark A. R. de Geus, Michael von Bergwelt-Baildon, Dominik Schumacher, Jonas Helma, Christian P. R. Hackenberger, Katharina S. Götze, Irmela Jeremias, Heinrich Leonhardt, Michaela Feuring, Karsten Spiekermann

**Affiliations:** 1 Department of Medicine III, LMU University Hospital, LMU Munich, Munich, Germany; 2 Chemical Biology, Leibniz-Forschungsinstitut für Molekulare Pharmakologie, Campus Berlin, Berlin, Germany; 3 Department of Chemistry, Humboldt Universität zu Berlin, Berlin, Germany; 4 Tubulis GmbH, Munich, Germany; 5 German Cancer Consortium (DKTK), partner site Munich, a partnership between DKFZ and LMU University Hospital, Munich, Germany; 6 Research Unit Apoptosis in Hematopoietic Stem Cells (AHS), Helmholtz Munich, German Research Center for Environmental Health (HMGU), Munich, Germany; 7 Faculty of Biology, Human Biology and BioImaging, LMU Munich, Planegg-Martinsried, Germany; 8 Department of Internal Medicine III, University Hospital Ulm, Ulm, Germany; 9 German Cancer Research Center (DKFZ), Heidelberg, Germany; 10 Technical University of Munich School of Medicine and Health, Department of Medicine III, Technical University of Munich (TUM), Munich, Germany; 11 Bavarian Cancer Research Center (BZKF), Munich, Germany; 12 Department of Pediatrics, Dr. von Hauner Children’s Hospital, LMU University Hospital, LMU, Munich, Germany

**Keywords:** Targeted therapies, Cancer stem cells, Acute myeloid leukaemia

## Abstract

Refractory disease and relapse are major challenges in acute myeloid leukemia (AML) therapy attributed to survival of leukemic stem cells (LSC). To target LSCs, antibody-drug conjugates (ADCs) provide an elegant solution, combining the specificity of antibodies with highly potent payloads. We aimed to investigate if FLT3-20D9h3-ADCs delivering either the DNA-alkylator duocarmycin (DUBA) or the microtubule-toxin monomethyl auristatin F (MMAF) can eradicate quiescent LSCs. We show here that DUBA more potently kills cell-cycle arrested AML cells compared to microtubule-targeting auristatins. Due to limited stability of 20D9h3-DUBA ADC in vivo, we analyzed both ADCs in advanced in vitro stem cell assays. 20D9h3-DUBA successfully eliminated leukemic progenitors in vitro in colony-forming unit and long-term culture initiating cell assays, both in patient cells and in patient-derived xenograft (PDX) cells. Further, it completely prevented engraftment of AML PDX leukemia-initiating cells in NSG mice. 20D9h3-MMAF had a similar effect in engraftment assays, but a less prominent effect in colony assays. Both ADCs did not affect healthy stem and progenitor cells at comparable doses providing the rationale for FLT3 as therapeutic LSC target. Collectively, we show that FLT3-directed ADCs with DUBA or MMAF have potent activity against AML LSCs and represent promising candidates for further clinical development.

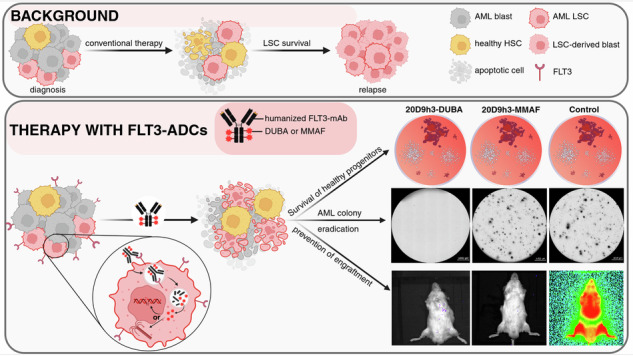

## Introduction

Around 50% of older and 20–40% of younger acute myeloid leukemia (AML) patients are refractory to induction therapy [1] and detection of minimal residual disease (MRD) after induction greatly increases chances of relapse [2]. There is considerable evidence, that leukemic stem cells (LSCs) and stemness signatures correlate with MRD and relapse risk [3, 4], and are prognostic for treatment success [5, 6]. Thus, finding ways to eliminate LSCs is of utmost importance. LSCs are characterized by their equal ability to self-renew and to differentiate [7, 8] and are the root of the hierarchically organized disease [9]. Several factors contribute to their drug resistance, e.g. quiescence [10], localization in the bone marrow niche [11] and expression of drug-resistance related genes [10]. There has been substantial effort in trying to identify specific LSC surface markers for targeted therapy. CD33, CD123, CLL-1, CD44 and CD47 have been reported among others [4, 10, 12]. Antibody-drug conjugates (ADCs) combine the specificity of antibodies with highly potent payloads and provide an elegant option for LSC-targeting. Several of such ADCs have been developed some of which claimed anti-LSC activity, like the FDA-approved CD33-directed gemtuzumab-ozogamicin (GO) [13], CLL-1-ADCs [14, 15] and CD123-ADCs [16–20] conjugated to payloads with different modes-of-action, spanning DNA-damaging agents, topoisomerase-I-inhibitors and kinesin-spindle-inhibitors. CD33 and CD123, however, are also expressed on healthy hematopoietic stem (HSC) and progenitor cells, thereby narrowing the therapeutic window; CLL-1 is expressed on AML bulk cells but shows low and variable expression on LSCs [21]. Fms-like tyrosine kinase 3 (FLT3) is present on a majority of AML bulk cells and LSCs regardless of disease stage, with low expression on a subset of healthy progenitors and solely cytoplasmic occurrence in other tissues [22–24]. FLT3 serves as target for various FDA-approved tyrosine kinase inhibitors [25–27] and other preclinical therapies, such as bispecific T-cell engagers [22, 23], chimeric antigen receptor T cells [28] and our recently presented 20D9-MMAF-ADC [29], applying the microtubule-targeting payload monomethyl auristatin F (MMAF). However, the effectiveness of FLT3-directed ADCs towards LSCs has not yet been addressed. Due to their quiescent phenotype, it is widely assumed that DNA-damaging rather than microtubule-targeting agents are more suitable to kill LSCs [14, 30, 31]. Still, excellent efficacy of MMAF-conjugates with long lasting complete responses in patient-derived xenograft (PDX) models could be shown by us and others [29, 32]. From the DNA-alkylator payloads, duocarmycin (DUBA) has recently shown potential as ADC payload in various cancer entities [30]. Here, we aimed to investigate if FLT3-ADCs utilizing DUBA or MMAF as payloads could efficiently eradicate LSCs. Therefore, we generated a humanized FLT3-directed 20D9h3-antibody and conjugated it to DUBA or MMAF. We could show that 20D9h3-DUBA has promising therapeutic activity against LSCs, but challenges remain in its stability. 20D9h3-MMAF had superior stability but lower activity towards LSCs.

## Methods

### Human samples

Primary samples from AML patients were collected by the Department of Internal Medicine III, LMU, Munich, Germany, and healthy bone marrow (BM) cells for colony-forming unit (CFU) assay by the Department of Internal Medicine III, TUM, Munich, Germany. Written informed consent was obtained from all patients. The studies were performed in accordance with the ethical standards of the responsible committees on human experimentation (written approval by Ethikkommission des Klinikums der Ludwig-Maximilians-Universität München, ethikkommission@med.uni-muenchen.de, number 068-08 and 222-10 and by Ethikkommission an der Technischen Universität München, ethikkommission@mri.tum.de, number 339/21S) and with the Helsinki Declaration of 1975, as revised in 2013. PDX cells were amplified in ten to 26-weeks-old male or female NOD.Cg-*Prkdc*^*scid*^ *Il2rg*^*tm1Wjl*^/SzJ (NSG, The Jackson Laboratory, Bar Harbour, Maine, USA) mice as described [33]. Animal trials were performed in accordance with current ethical standards of the official committee on animal experimentation (written approval by Regierung von Oberbayern, tierversuche@reg-ob.bayern.de, ROB-55.2Vet-2532.Vet 03-21-9). CD34-positive cells from BM were isolated as described [34]. CD34-positive cells for long-term culture initiating cell (LTC-IC) assays were purchased (STEMCELL Technologies, Vancouver, Canada).

### Treatment of AML PDX and normal BM cells

Healthy CD34-positive BM cells were cultivated in IMDM, GlutaMAX containing 10% BIT9500, 3 U/ml EPO, 20 ng/ml of IL-3, IL-6, G-CSF and GM-CSF and 100 ng/ml of FLT3L and SCF (all Immunotools, Friesoythe, Germany). AML PDX cells were either freshly isolated from BM of donor mice or thawed. PDX and primary cells were cultivated as described [35] additionally including 10 ng/ml FLT3L. For CFU and LTC-IC assays, we treated 5 × 10^4^ (CFU) and 1.7 × 10^4^ (LTC-IC) CD34-positive BM cells or 3 × 10^5^ (CFU) and 5 × 10^6^ (LTC-IC) AML PDX cells in total per condition, respectively. For retransplantation assays, we planned to inject pre-treated AML PDX cells at 100-time leukemia-initiating cell (LIC)-frequency; therefore, we plated 1 × 10^5^ and 5 × 10^4^ cells per mouse per condition for AML-388 and AML-393, respectively [36]. All cells were treated with 0.3–1 µg/ml isotype control ADCs (IgG1-DUBA/IgG1-MMAF) or 0.005–1 µg/ml FLT3-ADCs (20D9h3-DUBA/20D9h3-LALA-DUBA/20D9h3-MMAF) or left untreated. After 48-96 h, cells were collected and washed prior to plating them in the different assays.

### CFU and LTC-IC assays

After 4 d drug treatment, CFU assay for healthy CD34-positive cells was performed as described [29]. AML PDX and primary AML samples were plated in MethoCult H4034 (STEMCELL Technologies) in duplicates as described [37]. After 14 d (37 °C, 5% CO_2_), we screened for clusters (20-50 cells) and colonies ( > 50 cells).

LTC-IC assay was performed as described in detail before [38, 39]. Briefly, after 48 h drug treatment, all cells were collected and plated on feeder layers for 5 weeks with weekly half-medium changes in Myelocult H5100 (STEMCELL Technologies) including 1 µM hydrocortisone (added weekly) and 50 ng/ml SCF (added half-weekly). Feeder layers of irradiated (8000 cGy) M2-10B4 (CD34-positive BM cells) or SLSL-J-IL3-neo (PDX) murine fibroblasts were established beforehand in collagen-coated plates. After 5 weeks, cells were harvested, re-suspended in IMDM + 2% FBS and plated in methylcellulose as described for CFU assay. Healthy CD34-positive cells were plated in MethoCult H4230 (STEMCELL Technologies) with 3 U/ml EPO, 50 ng/ml SCF and 20 ng/ml IL-3, IL-6, GM-CSF and G-CSF; PDX cells were plated in MethoCult H4034.

### Retransplantation assay

After 4 d drug treatment, PDX cells were resuspended in PBS, and retransplanted into groups of mice by tail vein injection (*n* = 5 per group). Engraftment was monitored by repeated bioluminescence imaging (BLI).

Additional methods are provided in supplementary methods.

## Results

### Humanization of anti-FLT3 antibody 20D9

**Fig. 1:**
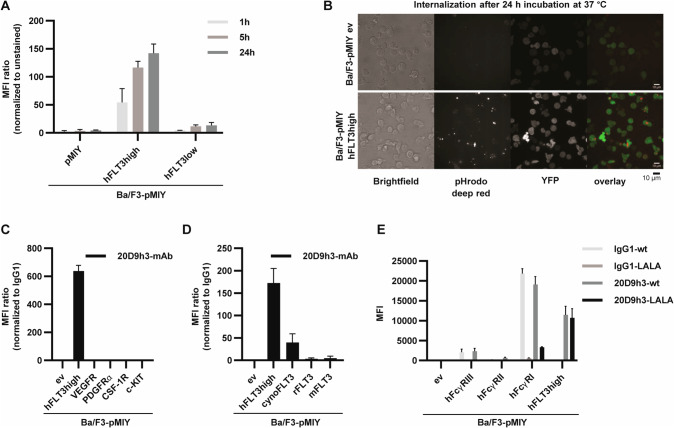
Characterization of binding and internalization properties of the humanized FLT3 monoclonal antibody 20D9h3. **A**, **B** 20D9h3 monoclonal antibody (mAb) was complexed with a pHrodo Deep Red-labelled secondary antibody and incubated with Ba/F3 cells stably expressing empty MSCV-IRES-YFP vector (pMIY ev) or human wildtype FLT3 at low (hFLT3low) or high (hFLT3high) surface levels. Internalization was measured by flow cytometry after 1 h, 5 h and 24 h depicted as mean fluorescence intensity (MFI) ratio to unstained control (**A**) or by microscopy after 24 h (**B**). Green = stably expressed YFP, red = 20D9h3-mAb visualized by pHrodo-labelled secondary antibody. Scale bar = 10 µm; Nikon CFI Apochromat TIRF 60x NA 1.49 oil immersion objective. mean±s.d.; *n* = 3. Binding of 20D9h3-mAb to Ba/F3-pMIY cells expressing ev, hFLT3high, FLT3 paralogues (VEGFR, PDGFRα, CSF-1R and c-KIT) (**C**) or FLT3 orthologues (to cynomolgus monkey (cynoFLT3), rat (rFLT3) and mouse (mFLT3) FLT3) (**D**) measured by flow cytometry after 1 h incubation. MFI was normalized to binding of IgG1 isotype control. mean±s.d.; *n* = 3. **E** Binding of 20D9h3-mAb and IgG1 isotype control with wildtype (wt) or Leu234Ala/Leu235Ala (LALA)-mutated Fc domain to Ba/F3-pMIY cells expressing ev, immunoglobulin gamma Fc receptor (FcγRI, FcγRII, FcγRIII) or hFLT3high measured by flow cytometry after 1 h incubation and depicted as MFI. mean±s.d.; *n* = 4.

We recently generated an anti-FLT3 chimeric monoclonal antibody (mAb) 20D9 [29], which we now further improved by humanization using CDR grafting and a 3D-structure based approach. This resulted in four sequences of heavy (V_H_1-V_H_4) and light (V_L_1-V_L_4) chain variable domains, respectively, which were cloned into mammalian IgG expression vectors (Supplementary Fig. 1A). Of 16 possible antibodies, only those with V_L_1-V_L_3 could be expressed, resulting in 12 mAbs, which were extensively analyzed regarding affinity and stability (Supplementary Fig. 1B, C, Supplementary Table 1) to find an ideal candidate for ADC development. Overall, mAbs with V_L_1 (20D9h1-20D9h4) best preserved the high binding affinity to human FLT3 of 20D9 with half maximal effective concentrations (EC_50_) between 11.0–11.4 ng/ml versus 29.3–111.6 ng/ml for all other mAbs. Similarly, those mAbs showed highest binding to Ba/F3 cells stably expressing human wildtype FLT3 (hFLT3high), and no binding to control cells expressing no surface antigen (pMIY ev). Based on those criteria together with good expression yield and excellent degree of humanization we selected clone 20D9h3 for further characterization. Internalization constitutes a key-attribute for antibodies to be applied to ADCs [40]. We analyzed 20D9h3 internalization with microscopy and flow cytometry in Ba/F3-pMIY hFLT3 cells with high or low FLT3 expression using a pH-sensitive dye (Fig. 1A, B, expression in Supplementary Fig. 1D). 20D9h3 is rapidly internalized in FLT3-expressing cells with intracellular presence already after 1 h and dependent on FLT3 surface expression levels. Next, we tested the binding of 20D9h3 to paralogues of FLT3 receptor (Fig. 1C, expression in Supplementary Fig. 1E), which were selected based on sequence similarity and 3D-structure homology. 20D9h3 specifically bound Ba/F3-pMIY cells expressing FLT3, but not VEGFR, PDGFRα, CSF-1R, and c-KIT. 20D9h3 bound cynomolgus monkey (cynoFLT3), but not murine (mFLT3) or rat (rFLT3) FLT3 (Fig. 1D, expression in ref. [29] and Supplementary Fig. 1F). We recently showed that dual-targeting of FLT3 and immunoglobulin gamma Fc receptor I (hFcγRI) via the antibody IgG1 backbone with 20D9-ADC was superior compared to targeting FLT3 alone in vitro [29]. To investigate the role of targeting both receptors in eliminating LSCs, we generated 20D9h3- and IgG1-mAbs with Leu234Ala/Leu235Ala (LALA)-mutated Fc part [41] to ablate FcγR binding. LALA-mutation completely eliminated binding to Ba/F3-pMIY cells expressing FcγRIII (hFcγRIII) and strongly reduced binding to hFcγRI. We did not observe binding to FcγRII (hFcγRII) for mAbs with both wildtype and LALA-mutated Fc (Fig. 1E, expression in ref. [29]). Taken together, we identified humanized FLT3-mAb 20D9h3 as suitable candidate for ADC development.

### Activity of different payloads towards non-proliferating cells

**Fig. 2:**
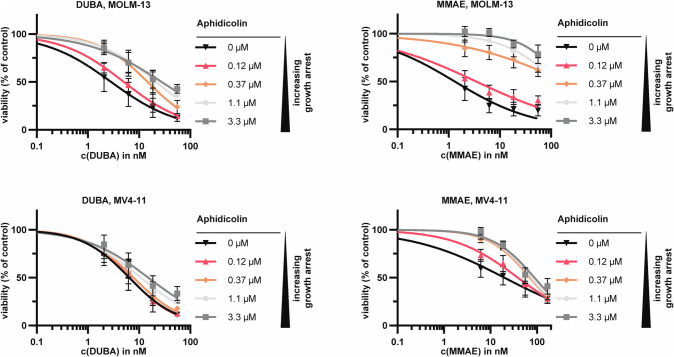
Selection of a payload to target resting cells. MOLM-13 and MV4-11 cells were treated with 0.12 µM-3.3 µM of aphidicolin or with vehicle-control (dimethyl sulfoxide = DMSO) for 24 h. Then, different concentrations of duocarmycin (DUBA) or monomethyl auristatin E (MMAE) were added for another 24 h. Viable cells were subsequently assessed with resazurin assay and are depicted normalized to the respective aphidicolin-only control. mean±s.d.; *n* = 4 biological replicates.

Current ADCs are mainly equipped with toxins from three classes: Microtubule-toxins (e.g. monomethyl auristatin E and F (MMAE/MMAF)), DNA-damaging agents, including DNA-alkylators (e.g. duocarmycin (DUBA)/Pyrrolobenzodiazepines), and topoisomerase-I-inhibitors (e.g. SN38/Dxd/exatecan). We tested drugs from all classes on four leukemia cell lines and Ba/F3 cells (Supplementary Fig. 2A). Overall, DUBA was most efficient with 50% inhibitory concentration values (IC_50_) between 0.58-21.25 nM. In K-562 cells, which were generally more resistant to all drugs, DUBA was highly effective (IC_50_ = 21.25 nM versus >790 nM for all other drugs). To target quiescent LSCs, the payload needs to be able to kill non-proliferating cells. In order to mimic a resting state, we treated MOLM-13 and MV4-11 cells with aphidicolin for 24 h. This leads to G1-arrest and completely inhibits proliferation at concentrations ≥ 0.37 µM without affecting viability (Supplementary Fig. 2B, C). Subsequently, we added payloads from all classes and performed cytotoxicity assays (Fig. 2, Supplementary Fig. 2D). In MOLM-13 cells the IC_50_ values of MMAE at 3.3 µM and 0 µM aphidicolin differed by a factor of 127.7 while for DUBA and exatecan the difference was smaller (9.8× and 55.4× for DUBA and exatecan, respectively). Similarly in MV4-11 the differences in IC_50_ values between 3.3 µM and 0 µM aphidicolin were largest for MMAE (4.4×), followed by exatecan (3×) and DUBA (2.4×) (Supplementary Table 2). MMAE has been used in this setting since membrane-impermeable MMAF only shows potency when actively delivered into the targeted cell, but not as payload alone. Due to these results and considering that topoisomerase-I-inhibitors are mostly used as payload class in solid tumor indications, we considered DUBA to be a good candidate for an LSC-targeting ADC and continued with the analysis of DUBA- and MMAF-conjugated ADCs based on 20D9h3.

### Generation and in vitro cytotoxicity evaluation of 20D9h3-DUBA and 20D9h3-MMAF

**Fig. 3:**
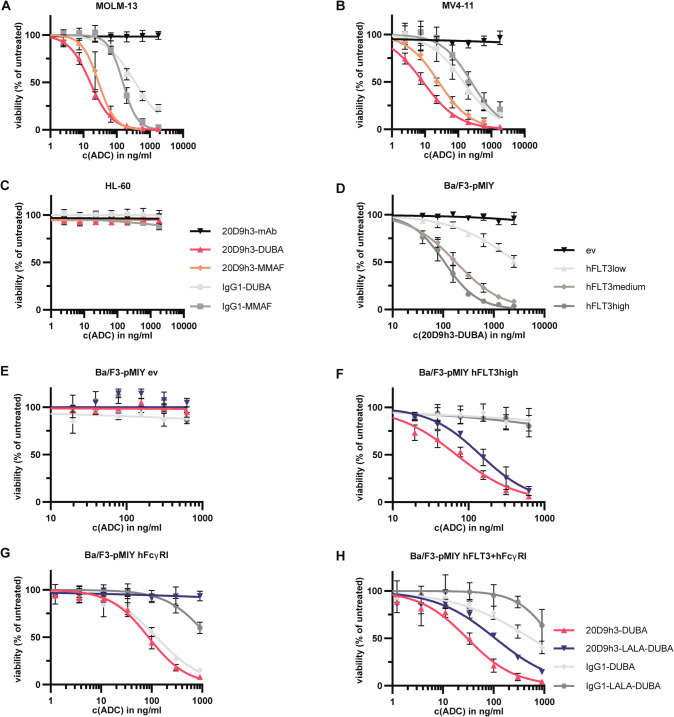
In vitro cytotoxicity evaluation of 20D9h3-DUBA in comparison to 20D9h3-MMAF. MOLM-13 (FLT3-positive, **A**), MV4-11 (FLT3-positive, **B**) and HL-60 (FLT3-negative, **C**) cells were treated with a dilution row of 20D9h3-mAb, 20D9h3-DUBA, 20D9h3-MMAF, IgG1-DUBA or IgG1-MMAF for 96 h. Viable cells were assessed by resazurin readout and normalized to untreated control. mean±s.d; *n* = 3 biological replicates. **D** Ba/F3-pMIY cells expressing ev, hFLT3low, hFLT3medium or hFLT3high were treated with different concentrations of 20D9h3-DUBA for 72 h. Viable cells were assessed by trypan blue exclusion and normalized to untreated control. mean±s.d.; *n* = 3 biological replicates. Ba/F3-pMIY cells expressing ev (**E**), hFLT3high (**F**), FcγRI (**G**) or hFLT3+FcγRI (**H**) were treated with a dilution row of 20D9h3-DUBA, 20D9h3-LALA-DUBA, IgG1-DUBA or IgG1-LALA-DUBA for 72 h. Viable cells were assessed by flow cytometry using LIVE/DEAD fixable far red dead cell stain and normalized to untreated control. mean±s.d.; *n* = 3 biological replicates.

Next, we generated 20D9h3-DUBA ADC with a drug-to-antibody ratio (DAR) of 4.8 by maleimide-cysteine-conjugation [42] and 20D9h3-MMAF ADC with a DAR of 8.0 by P5-technology [43–45]. It should be noted that 20D9h3-DUBA exhibited a strong tendency to aggregate, even though conjugated to less payload molecules, whereas 20D9h3-MMAF showed highest homogeneity. Irrespective of the lower DAR, 20D9h3-DUBA had quite similar cytotoxicity compared to 20D9h3-MMAF in FLT3-positive MOLM-13, MV4-11 and OCI-AML3 cells (Fig. 3A, B, Supplementary Fig. 3A, IC_50_ values in Supplementary Table 3). FLT3-negative K-562 and HL-60 cells were not affected by both ADCs (Fig. 3C, Supplementary Fig. 3B). Efficacy was dependent on FLT3 surface expression levels as demonstrated for 20D9h3-DUBA (Fig. 3D). Moreover, cytotoxicity was specific for FLT3 and FLT3 paralogues did not trigger cytotoxicity (Supplementary Fig. 3C, D). Next, we generated DUBA-conjugates from the LALA-mutated 20D9h3 and IgG1 isotype control. Neither of the four ADCs affected Ba/F3-pMIY ev cells (Fig. 3E, Supplementary Table 4). As expected from the binding studies, both 20D9h3-DUBA and 20D9h3-LALA-DUBA were similarly effective in eliminating Ba/F3-pMIY hFLT3 cells with IC_50_ values of 69.3 ng/ml and 142.9 ng/ml, respectively, whereas IgG1-ADCs were ineffective in those cells (Fig. 3F). Further, 20D9h3-DUBA and IgG1-DUBA were cytotoxic to hFcγRI-expressing cells, whereas their LALA-mutated versions had no effect (Fig. 3G). In Ba/F3-pMIY cells expressing both receptors (hFcγRI + hFLT3), the cytotoxic effect was: 20D9h3-DUBA > 20D9h3-LALA-DUBA > IgG1-DUBA > IgG1-LALA-DUBA (Fig. 3H).

### Mechanistic evaluation of 20D9h3-DUBA and 20D9h3-MMAF

**Fig. 4:**
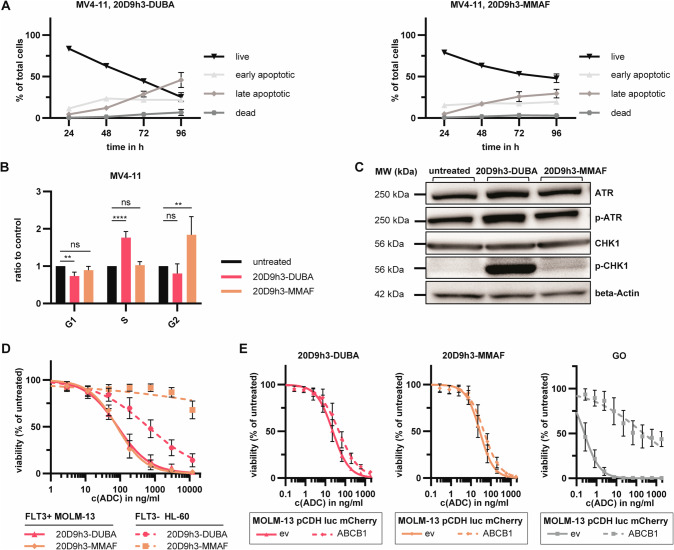
Mechanistic evaluation of 20D9h3-DUBA and 20D9h3-MMAF. **A** MV4-11 cells were treated with 100 ng/ml 20D9h3-DUBA (left) or 20D9h3-MMAF (right) for 24-96 h. Annexin V-positive and DAPI-positive cells were assessed every 24 h by flow cytometry and categorized as live cells (double negative), early apoptotic (Annexin V-positive), late apoptotic (double positive) or dead cells (DAPI-positive). mean±s.d.; *n* = 4 biological replicates. **B** MV4-11 cells were treated with 100 ng/ml 20D9h3-DUBA or 20D9h3-MMAF for 48 h. Cells were fixed with 80% (v/v) EtOH and stained with PI to evaluate cell cycle phase. The percentage of cells in G1, S and G2 was calculated with FlowJo version 10.8.1 using the Watson pragmatic algorithm. Values are depicted as ratio to untreated control. mean±s.d.; *n* = 4 biological replicates. **P < 0.01; ****P < 0.0001; ns, not significant by One-way ANOVA; each *p* value is adjusted to account for multiple comparisons. **C** MOLM-13 cells were treated with 50 ng/ml 20D9h3-DUBA or 20D9h3-MMAF or left untreated for 24 h. Afterwards, ATR, p-ATR, CHK1 and p-CHK1 expression was assessed by Western blotting using beta-Actin as loading control. Blots are representative of three independent experiments. MW = molecular weight. **D** FLT3-positive MOLM-13 cells stably expressing luciferase (luc) and mCherry and FLT3-negative native HL-60 cells were co-cultured at a ratio of 8:1 for 5 d in the presence of different doses of 20D9h3-DUBA or 20D9h3-MMAF. Viable cells were counted by flow cytometry using LIVE/DEAD™ Fixable Aqua Dead Cell Stain and normalized to untreated control. The two cell types were distinguished by mCherry signal. mean±s.d.; *n* = 3 biological replicates. **E** MOLM-13 luc mCherry cells with empty vector (ev) or ABCB1 expression were treated with a dilution row of 20D9h3-DUBA, 20D9h3-MMAF or GO for 96 h. Viable cells were assessed with resazurin readout and were normalized to untreated control. mean ± s.d.; *n* = 3 biological replicates.

We next investigated in more detail how 20D9h3-DUBA and 20D9h3-MMAF eliminate leukemic cells. We observed a robust induction of apoptosis by 20D9h3-DUBA in MV4-11 and MOLM-13 cells being very similar to 20D9h3-MMAF (Fig. 4A, Supplementary Fig. 4A). 20D9h3-DUBA arrested MV4-11 cells in G1/S, whereas 20D9h3-MMAF inhibited cells in G2/M (Fig. 4B). In contrast to 20D9h3-MMAF, 20D9h3-DUBA activated ATR-CHK1 DNA repair pathway in MOLM-13 cells indicating DNA damage (Fig. 4C). Further, 20D9h3-DUBA but not 20D9h3-MMAF induced bystander killing of FLT3-negative HL-60 cells, when co-incubated with FLT3-positive MOLM-13 or OCI-AML3 cells in the presence of ADC (Fig. 4D, Supplementary Fig. 4B). LSC treatment resistance was shown to be partly mediated by drug transporters e.g. ATP-dependent translocase (ABCB1) [12], as described for calicheamicin, the payload of FDA-approved CD33-targeting GO. 20D9h3-DUBA and 20D9h3-MMAF, however, seem to be no substrate of ABCB1 as their efficacy was not reduced in ABCB1-overexpressing MOLM-13 cells. (Fig. 4E, Supplementary Fig. 4C).

### Stability and pharmacokinetics of 20D9h3-DUBA and 20D9h3-MMAF

During ADC conjugation we observed a high aggregation tendency for 20D9h3-DUBA but not 20D9h3-MMAF (Supplementary Fig. 8, 9). Furthermore, the loss of linker-payload to serum proteins by thiol-exchange reaction is a known phenomenon of maleimide-cystein conjugation technology which was applied in 20D9h3-DUBA [46]. Additionally, it has been reported [47], that ADCs with the linker-drug vc-*seco*-DUBA are unstable specifically in rodent but not human plasma due to cleavage of the linker by murine carboxylesterase 1c (CES1c). In contrast, 20D9h3-MMAF is conjugated by P5-technology, which has been shown previously to exert excellent stability in vitro and in vivo [43–45]. We could confirm that 20D9h3-DUBA rapidly loses linker-payload when incubated ex vivo in mouse serum (Supplementary Fig. 10). We also observed retro-Michael related loss of the whole linker-payload via mass spectrometry; CES1c-mediated payload loss from the linker could not be confirmed. To investigate how this impacts pharmacokinetics, we injected each ADC in NSG mice and analyzed serum at different time points (Supplementary Fig. 11). Indeed, we observed by ELISA that intact 20Dh3-DUBA ADC levels rapidly decreased, whereas 20D9h3-MMAF proved to be stable. Taken together, the poor pharmacokinetics of 20D9h3-DUBA in mice is especially problematic for a low expressed target like FLT3 and can highly underestimate its anti-tumor effect. Hence, we continued our efforts with in vitro experiments and engraftment assays as we were mainly interested in analyzing the ADCs capability to kill LSCs.

### Elimination of short-term leukemic progenitors in vitro

**Fig. 5:**
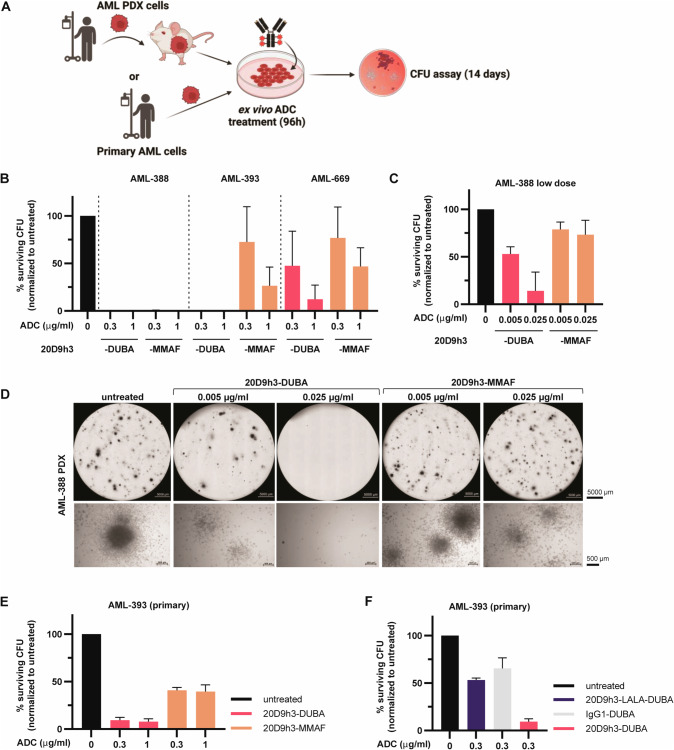
Evaluation of short-term AML progenitors in vitro. **A** Schematic description of colony-forming unit (CFU) assay. AML PDX cells, isolated from NSG donor mice, or primary AML cells were treated with the different ADCs for 96 h in vitro/ex vivo. Subsequently, cells were washed and plated in methylcellulose and colonies were counted after 14 d. Figure was created with BioRender.com and Adobe Illustrator, in parts modified from STEMCELL Technologies. **B**–**D** AML-388, AML-393 and AML-669 PDX cells (3 × 10^5^ cells per condition, respectively) were treated with indicated concentrations of ADCs for 96 h. On day 4, all remaining cells were plated in methylcellulose for CFU assay in technical duplicates without further treatment and incubated for 14 d. On day 14, cell clusters ( > 20 cells) and colonies ( > 50 cells) were counted and normalized to untreated control. mean±s.d.; AML-393 and AML-669 (**B**): *n* = 3 biological replicates; AML-388 (**B**–**D**): *n* = 2 biological replicates, each including two technical replicates. Quantification of colony numbers (**B**, **C**) and representative pictures of the whole well (scale bar = 5000 µm; PlanApo 2 × 0.10/8.50 mm objective) and zoom-ins (scale bar = 500 µm; Plan-ACHROMAT 4x/0.10 Ph0 objective) (**D**) are displayed. **E**, **F** Primary cells corresponding to AML-393 PDX (3 × 10^5^ cells per condition) were treated with 0.3 or 1 µg/ml 20D9h3-DUBA or 20D9h3-MMAF (**E**) or 0.3 µg/ml IgG1-DUBA or 20D9h3-LALA-DUBA (**F**) for 96 h. On day 4, all remaining cells were plated in methylcellulose in technical duplicates without further treatment, incubated for 14 d and colonies were counted as described before. mean±s.d.; *n* = 2 technical replicates.

Therefore, we next examined the activity of both ADCs towards AML leukemic progenitors and selected three PDX samples—AML-388, AML-393 and AML-669—with *KMT2A*-rearrangement (*KMT2Ar*) (Supplementary Table 5) for further experiments. We chose *KMT2Ar*-leukemias as they are known for their stem-cell like characteristics [48]. KMT2A fusion proteins act highly transformative and are often used to initiate leukemia with a high frequency of self-renewing cells [49] being a valuable model to study LSCs [48]. We have shown recently that all three PDX models express wildtype FLT3 at high levels [29]. First, we tested the general sensitivity of *KMT2Ar*-PDX cells in a 96 h cytotoxicity assay. In general, PDX cells were more sensitive towards 20D9h3-DUBA than 20D9h3-MMAF with AML-388 being especially sensitive towards both ADCs (Supplementary Fig. 5A). Next, we evaluated the survival of short-term AML progenitors in CFU assay (Fig. 5A). 20D9h3-DUBA was more effective than 20D9h3-MMAF to reduce the number of colony-forming cells (CFCs) in all PDX samples (Fig. 5B, Supplementary Fig. 5B, C, colony counts in Supplementary Table 6). AML-388 and AML-393 CFCs were completely eradicated by 0.3 µg/ml 20D9h3-DUBA, AML-669 CFC were reduced to 47.5% ± 36.4% of untreated control. At the same dose, 20D9h3-MMAF reduced CFC to 0.5% ± 0.5%, 72.7% ± 37% and 76.9% ± 32.5% for AML-388, AML-393 and AML-669, respectively. For AML-388, 20D9h3-DUBA reduced CFCs highly effective even at doses as low as 0.025 µg/ml (Fig. 5C, D). To ensure that the results with PDX cells reflect the situation in the patient, we repeated the assay with the primary patient sample that had been used to generate AML-393 PDX cells. We observed quite similar results, with 20D9h3-DUBA outperforming 20D9h3-MMAF (Fig. 5E). CFCs were reduced to 9.4% ± 3.1% versus 41% ± 3.1% at an ADC dose of 0.3 µg/ml, respectively. Further, we analyzed if co-targeting of FLT3 and FcγRI via the IgG1 antibody backbone is superior to targeting FLT3 or FcγRI alone. In AML-393 primary cells, targeting of FLT3 or FcγRI alone with 20D9h3-LALA-DUBA or IgG1-DUBA, reduced CFC to 53.2% ± 2.0% and 65.5% ± 11.2%, respectively. However, co-targeting of both receptors reduced AML-393 CFC to 9.4% ± 3.1% (Fig. 5F).

### Elimination of long-term leukemic progenitors in vitro and prevention of PDX engraftment in vivo

**Fig. 6:**
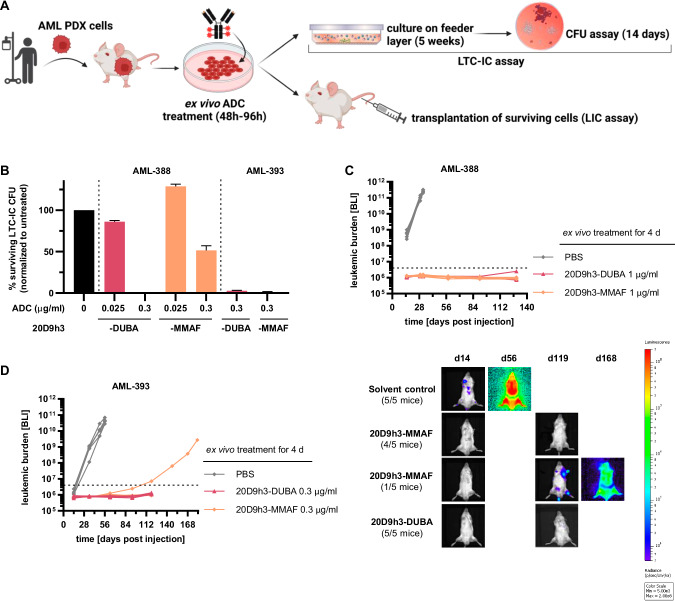
Evaluation of long-term AML progenitors in vitro and in vivo. **A** Schematic description of long-term culture initiating cell (LTC-IC) and leukemia-initiating cell (LIC) assay. AML PDX cells, isolated from NSG donor mice were treated with the different ADCs for 48-96 h in vitro/ex vivo (as individually stated). For LTC-IC assay, cells were then cultivated on a feeder layer of murine fibroblasts for 5 weeks and afterwards plated in methylcellulose for colony analysis on day 14. For LIC assay, cells were transplanted into NSG mice and engraftment was monitored by bioluminescence imaging (BLI). Figure was created with BioRender.com and Adobe Illustrator, in parts modified from STEMCELL Technologies. **B** AML PDX cells of AML-388 and AML-393 (5 × 10^6^ cells per condition, respectively) were treated with 0.025 µg/ml or 0.3 µg/ml 20D9h3-DUBA or 20D9h3-MMAF for 48 h. All remaining cells were harvested and co-cultured with irradiated SLSL-J-IL3-neo murine fibroblasts for 5 weeks without further treatment, then they were plated in methylcellulose in technical duplicates and incubated for 14 d. In analogy to the CFU assay, cell clusters ( > 20 cells) and colonies ( > 50 cells) were counted and normalized to untreated control. mean±s.d.; *n* = 2 technical replicates. Luc-positive mCherry-positive AML PDX cells of AML-388 (1 × 10^5^ cells per condition and mouse, (**C**) and AML-393 (5 × 10^4^ cells per condition and mouse, (**D**) were treated ex vivo with indicated concentrations of 20D9h3-DUBA or 20D9h3-MMAF for 96 h. On day 4, remaining cells were injected into ten-weeks old (AML-393) or 26-weeks-old (AML-388) male NSG recipient mice and engraftment was repeatedly monitored by BLI for up to 181 d. Right side: BLI image of one representative mouse per treatment group for AML-393 PDX cells. mean±s.d.; *n* = 5 mice per group. Dashed line = detection limit.

We next investigated the efficacy of both ADCs in LTC-IC assays in vitro and in engraftment assays in vivo for two of the AML PDX samples (Fig. 6A). In contrast to CFU assay, LTC-IC assay reflects the very primitive stem cell population [38, 50] that is functionally and phenotypically similar to in vivo LICs. Consistent with the CFU assay results, 20D9h3-DUBA was highly effective in LTC-IC assays (Fig. 6B, Supplementary Fig. 6A, colony counts in Supplementary Table 6). It reduced AML-388 LTC-IC-derived CFC (LTC-IC-CFC) to 0% ± 0% (0.3 µg/ml) and 86.1% ± 1.4% (0.025 µg/ml) of untreated control, respectively, and AML-393 LTC-IC-CFC to 2.8% ± 0.6% (0.3 µg/ml). 20D9h3-MMAF had significant activity in AML-393 cells (1.2% ± 0.6% LTC-IC-CFC at 0.3 µg/ml), but was less effective in AML-388 cells (128.7% ± 2.8% LTC-IC-CFC at 0.025 µg/ml and 51.5% ± 5.6% LTC-IC-CFC at 0.3 µg/ml). Next, we analyzed the effect of drug treatment on engraftment capability of AML PDX cells in NSG mice. We started by treating AML-388 cells with a high dose of 1 µg/ml ex vivo. In accordance with the in vitro results, AML-388 cells treated with either of the ADCs did not engraft, in contrast to PBS-treated cells which rapidly engrafted and showed a very aggressive disease course (Fig. 6C, Supplementary Fig. 6B). We repeated the experiment with a dose of 0.3 µg/ml at which 20D9h3-DUBA has been superior to 20D9h3-MMAF in CFU assays for AML-393. While 20D9h3-DUBA prevented AML-393 engraftment in all mice, one of the five mice of the 20D9h3-MMAF group showed a late positive outgrowth (Fig. 6D).

### Cytotoxicity towards healthy CD34-positive cells

**Fig. 7:**
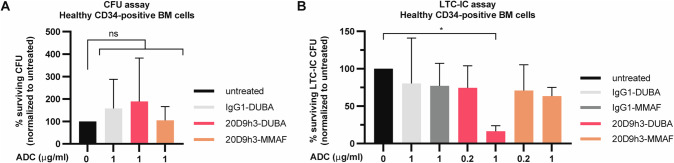
Evaluation of 20D9h3-DUBA toxicity towards healthy short and long-term progenitors in vitro. **A** CD34-positive BM cells from healthy human donors (5 × 10^4^ cells per condition) were treated with 1 µg/ml 20D9h3-DUBA, 20D9h3-MMAF or IgG1-DUBA for 96 h. On day 4, all remaining cells were plated in methylcellulose without further treatment and total colony counts were evaluated after 14 d. Counts were normalized to untreated control. mean±s.d.; *n* = 5 donors (except 20D9h3-MMAF group: *n* = 4 donors). ns, not significant by Kruskal-Wallis test; each *p* value is adjusted to account for multiple comparisons. **B** CD34-positive BM cells from healthy human donors were treated with 0.2 and 1 µg/ml 20D9h3-DUBA or 20D9h3-MMAF or 1 µg/ml IgG1-DUBA or IgG1-MMAF for 48 h. Remaining cells were harvested and cultured on a layer of irradiated M2-10B4 murine fibroblasts for 5 weeks without further treatment. After 5 weeks, all cells were harvested and plated in methylcellulose. Total colony numbers were assessed after 14 d and normalized to untreated control. mean±s.d.; *n* = 3 donors, each including two technical replicates. *P < .05 by One-way ANOVA; each *p* value is adjusted to account for multiple comparisons.

Next, we asked if doses effective against AML progenitors, would also harm healthy progenitors using colony assays with healthy CD34-positive BM cells (donor information in Supplementary Table 7). FLT3 and FcγRI were expressed on 48% and 8.1% of CD34-positive cells, respectively (Supplementary Fig. 7A). 1 µg/ml of 20D9h3-DUBA or 20D9h3-MMAF had only minor effects on the number of healthy progenitor CFCs compared to untreated control (Fig. 7A). Further, there was no difference in any particular type of progenitor between drug-treated and untreated samples (Supplementary Fig. 7B, C). Looking at the primitive progenitors in LTC-IC assay, we observed a significant reduction of LTC-IC-CFCs from CD34-positive BM cells (to 16.4% ± 7.4% of control) treated with 1 µg/ml of 20D9h3-DUBA (Fig. 7B). At a lower dose of 0.2 µg/ml that has been effective against AML progenitors, however, healthy progenitors were only slightly affected by 20D9h3-DUBA (reduction to 74.5% ± 29.4% of control). The isotype control ADC IgG1-DUBA decreased LTC-IC-CFC to 80.5% ± 60.4% of untreated control at 1 µg/ml. In contrast to 20D9h3-DUBA, 20D9h3-MMAF decreased healthy LTC-IC-CFCs to a lower extent, more specifically to 63.4% ± 11.6% and 70.8% ± 34.7% at 1 µg/ml and 0.2 µg/ml, respectively. Looking at the types of progenitors, 20D9h3-DUBA mostly reduced CFU-GM and CFU-M. 20D9h3-MMAF only exerted a minor adverse effect on CFU-GM (Supplementary Fig. 7D). Taken together, both ADCs only slightly affected healthy stem and progenitor cells at doses effective in leukemic cells.

## Discussion

Here, we report the analysis of two payloads, the microtubule-inhibitor MMAF and the DNA-alkylator DUBA conjugated to humanized FLT3-antibody 20D9h3 and compare their activity towards AML LSCs. We demonstrate that DUBA compared to MMAE (as the membrane-permeable surrogate of MMAF) efficiently killed G1-arrested MOLM-13 and MV4-11 cells indicating superiority of DNA-damage over microtubule-targeting in addressing non-proliferating cells. Furthermore, 20D9h3-DUBA ADC activates ATR-CHK1 DNA-damage repair pathway confirming its DNA-damaging activity. 20D9h3-DUBA shows similar or superior cytotoxicity towards leukemia cell lines and PDX cells compared to 20D9h3-MMAF despite the lower DAR of 4.8 versus 8.0. The promising in vitro results with 20D9h3-DUBA are currently challenging to translate to in vivo due to stability issues of the linker-payload. We observed a strong aggregation tendency of 20D9h3-DUBA. Moreover, pharmacokinetic analyses revealed that 20D9h3-DUBA rapidly loses payload in circulation. Retro-Michael addition of the maleimide in the linker of 20D9h3-DUBA and the mouse-specific enzyme CES1c are likely leading to this pharmacokinetic profile. Further linker optimization of 20D9h3-DUBA is crucial to solve these issues, which are currently preventing further preclinical developments. These optimizations are out of scope since the aim of this study was to answer pressing questions on which payload to use for LSC eradication.

Hence, a key question around the modes of action of the two payloads in the context of eradicating LSCs when delivered via FLT3 could be answered in extensive CFU and LTC-IC assays with AML PDX cells in vitro. Here, 20D9h3-DUBA was more active in the eradication of leukemic progenitors compared to 20D9h3-MMAF. Furthermore, ex vivo treatment of AML-388 and AML-393 PDX with 20D9h3-DUBA completely prevented engraftment in NSG mice. In the 20D9h3-MMAF-treated AML-393 sample, one mouse showed a late engraftment, whereas for AML-388 all mice stayed negative—suggesting that 20D9h3-MMAF is also active towards LICs. This is surprising as—due to its mode-of-action—MMAF is generally not regarded as ideal ADC payload to target quiescent cells [14, 30, 31]. It is, however, in line with our previous findings where 20D9-MMAF led to sustained tumor reduction in NSG mice transplanted with AML-573 PDX cells indicating that LICs have been targeted [29]. Similarly, other studies have reported activity of microtubule-targeting agents towards stem cells [51–53]. One reason might be that tubulin-inhibition not only acts on cell division but also interferes with intracellular protein trafficking [54, 55]. Some genetic aberrations might further increase vulnerabilities for microtubule-toxins, e.g. *KMT2A/AF6* rearrangement has been reported to cause cytoskeletal abnormalities [56]. This might explain why 20D9h3-MMAF is very effective towards AML-388 cells, which carry a *KMT2A/AF6* rearrangement. Nevertheless, 20D9h3-DUBA was clearly more potent than 20D9h3-MMAF in cytotoxicity assays in vitro, especially considering the lower DAR, and eliminated PDX samples more effectively and at lower doses in colony assays.

FLT3 has been described as promising LSC target [12, 22–24]. We could show that FLT3-directed 20D9h3-DUBA efficiently eradicates leukemic stem and progenitor cells. Recently, we reported superiority of co-targeting FLT3 and FcγRI via the variable and constant domains of the antibody in vitro [29]. Similarly, co-targeting eliminated leukemic progenitors in CFU assay more efficiently compared to targeting FLT3 or FcγRI alone. Compared to other ADC targets, FLT3 expression is relatively low which might lead to challenges in the effectivity of FLT3-mediated targeted drug delivery [22]. Our data show that FLT3-targeting is generally feasible but requires a highly potent payload. Moreover, we did not observe substantial killing of healthy progenitors at doses sufficient to eradicate their leukemic counterparts. This provides rational to use FLT3 as target to specifically address leukemic but not healthy stem cells. This is further supported by literature, where FLT3-BITEs were shown to be safe in cynomolgus monkeys leading to only mild, partial and reversible decreases of certain FLT3-positive healthy hematopoietic progenitor subsets, dendritic cells, monocytes and B cells, and no non-hematological toxicities [22, 23]. Also, the phase I clinical evaluation of the FLT3-mAb FLYSYN had encouraging safety outcomes: it was well-tolerated with only mild to moderate mostly hematology associated side effects [57]. Toxicity evaluations for ADCs are more complex and influenced also by linker stability and nature of the payload and will require the investigation in relevant species such as non-human primates as a next step.

In conclusion, we describe the humanization and preclinical characterization of FLT3-directed ADCs utilizing the DNA-damaging agent DUBA or the microtubule-inhibitor MMAF. Our data indicate that using FLT3 as target and DUBA as payload efficiently eradicates leukemic progenitors in vitro and prevents LIC engraftment in vivo suggesting this combination as promising for an anti-LSC ADC. Further developments will include linker optimization of 20D9h3-DUBA by employing P5-technology [43–45]. The resulting candidate might combine the good biophysical properties of 20D9h3-MMAF with the excellent LSC-eradicating properties of the DUBA payload.

## Supplementary information

Supplemental methods, results, tables, figures and references.

## Data Availability

All data generated or analyzed during this study are included in this published article and its supplementary information files.
