# Supplementary material for: Effective eradication of acute myeloid leukemia stem cells with FLT3-directed antibody-drug conjugates
Source: Leukemia. 2025 Jan 27;39(3):632–42. doi: 10.1038/s41375-024-02510-5 (PMC11879846; doi:10.1038/s41375-024-02510-5)
Supplement: Supplementary file 1 — Supplemental methods, results, tables, figures and references. [file 41375_2024_2510_MOESM1_ESM.docx]

**Effective eradication of acute myeloid leukemia stem cells with FLT3-directed antibody-drug conjugates**

Marina Able^1^, Marc-André Kasper^2-4^, Binje Vick^5,6^, Jonathan Schwach^7^, Xiang Gao^8^, Saskia Schmitt^4^, Belay Tizazu^1^, Amrei Fischer^1,5,9^, Sarah Künzl^1^, Marit Leilich^10^, Isabelle Mai^4^, Philipp Ochtrop^2,4^, Andreas Stengl^7^, Mark A.R. de Geus^2^, Michael von Bergwelt-Baildon^1,5,11^, Dominik Schumacher^2-4^, Jonas Helma^4,7^, Christian P. R. Hackenberger^2,3^, Katharina S. Götze^5,10,11^, Irmela Jeremias^5,6,12^, Heinrich Leonhardt^7*^, Michaela Feuring^8*^ and Karsten Spiekermann^1,5,11*^

*^1^Department of Medicine III, LMU University Hospital, LMU Munich, Munich, Germany; ^2^Chemical Biology, Leibniz-Forschungsinstitut für Molekulare Pharmakologie, Campus Berlin, Berlin, Germany; ^3^Department of Chemistry, Humboldt Universität zu Berlin, Berlin, Germany; ^4^Tubulis GmbH, Munich, Germany; ^5^German Cancer Consortium (DKTK), partner site Munich, a partnership between DKFZ and LMU University Hospital, Munich, Germany; ^6^Research Unit Apoptosis in Hematopoietic Stem Cells (AHS), Helmholtz Munich, German Research Center for Environmental Health (HMGU), Munich, Germany; ^7^Faculty of Biology, Human Biology and BioImaging, LMU Munich, Planegg-Martinsried, Germany; ^8^Department of Internal Medicine III, University Hospital Ulm, Ulm, Germany; ^9^German Cancer Research Center (DKFZ), Heidelberg, Germany; ^10^Technical University of Munich School of Medicine and Health, Department of Medicine III, Technical University of Munich (TUM), Munich, Germany; ^11^Bavarian Cancer Research Center (BZKF); ^12^Department of Pediatrics, Dr. von Hauner Children's Hospital, LMU University Hospital, LMU Munich, Germany.*

*These authors contributed equally to this work.

Correspondence: Karsten Spiekermann, Medizinische Klinik und Poliklinik III, Marchioninistraße 15, 81377 München; e-mail: karsten.spiekermann@med.uni-muenchen.de

**Running title:** Killing of AML LSCs by FLT3-ADCs

**Table of contents**

[**Supplementary Methods** 3](#_Toc159858455)

[**Supplementary Tables** 17](#_Toc159858456)

[**Supplementary Figures** 21](#_Toc159858457)

[**Supplementary References**](#_Toc159858458) 36

#

# **Supplementary Methods**

**Antibody expression**

All antibodies were expressed using the ExpiCHO expression system (Thermo Fisher Scientific, Waltham, Massachusetts, US). CHO cells were subcultured to a maximum of 2x10^6^ cells/ml in a 125 ml shaking flask and incubated at 37 °C, 8% CO_2_, 180 rpm. Expression was performed using the standard expression protocol according to the manufacturer’s instructions. In brief: One day prior to transfection CHO cells were split to 3x10^6^ cells/ml and incubated overnight. On the day of transfection, cells were diluted to 6x10^6^ cells/ml in a volume of 25 ml. Transfection was performed with a total of 20 µg DNA (10 µg of heavy and light chain plasmid, respectively) diluted in 1 ml OptiPRO SFM (Thermo Fisher Scientific). In parallel, 80 µl of ExpiCHO transfection reagent were diluted in 920 µl OptiPRO SFM. Diluted DNA and transfection reagent were mixed and incubated for 3 min before addition to the CHO cells. One day after transfection, 6 ml of feed medium and 150 µl of enhancer reagent were added. Expression was typically performed for 7-10 d before harvest. If larger quantities of antibody were needed, all volumes were scaled by a factor of 2-3 and cells were cultured in an appropriately larger flask.

**Antibody purification via Protein A chromatography**

CHO cells were pelleted by centrifugation at 1 000 g for 10 min. Supernatant was harvested and sterile filtered using a 0.22 µm sterile filter. Purification was performed on an Äkta Pure System using a MabSelect Sure Protein A column (both Cytiva, Marlborough, Massachusetts, US). System and column were equilibrated in 5 CV mAb binding buffer (20 mM NaH_2_PO_4_, 50 mM NaCl, 1 mM EDTA, pH 7.5, sterile filtered) before the filtered supernatant was loaded onto the column at a flowrate of 0.5 ml/min. The column was subsequently washed at 1 ml/min with 10 CV of binding buffer. Elution was performed at 1 ml/min with 10 CV elution buffer (100 mM sodium citrate, pH 3.0, sterile filtered) and directly eluted into a prepared vessel containing neutralization buffer (1 M Tris-HCl, pH 9.0, sterile filtered) for neutralization to a final pH of 7-7.5. Fractions were collected, pooled and concentrated using Amicon® Ultra 0.5 ml Centrifugal Filters (Merck, Burlington, Massachusetts, US). Buffer was exchanged by 0.5 ml Zeba Spin desalting columns (Thermo Fisher Scientific, 7 MWCO) to storage buffer (100 mM L-arginine in PBS, pH 7.3, sterile filtered).

**Antibody stability test**

Antibodies were diluted to 1 mg/ml and 0.1% sodium azide were added to prevent bacterial growth. Samples were divided into three tubes each for incubation at 4 °C, 25 °C and 37 °C. After one, two and four weeks of incubation, respectively, samples for each condition were centrifuged at 10 000 g for 10 min prior to analysis by size-exclusion chromatography (SEC) and hydrophobicity interaction chromatography (HIC).

**Generation of Leu234Ala/Leu235Ala point mutations**

We generated Fc-binding silenced antibodies by introduction of Leu234Ala/Leu235Ala (LALA) point mutations. These point mutations were introduced by priming the adjacent region with oligonucleotides carrying overhangs for the LALA mutant and performing PCR with Phusion polymerase (Thermo Fisher Scientific; 5 min initial denaturation at 98 °C, 30 cycles of 20 s denaturation at 98 °C, 10 s annealing at 55 °C, 15 s elongation at 72 °C, final elongation 2 min at 72 °C). pcDNA3.420D9h3 vector was digested with FastDigest BamHI and FastDigest XagI restriction enzymes (both Thermo Fisher Scientific) to remove the unmutated sequence and cleaned up via agarose gel electrophoresis. Gibson Assembly (New England Biolabs, Ipswich, Massachusetts, US) was used to assemble the digested vector and PCR products according to the manufacturer’s instructions.

**Chemicals, solvents and antibodies for ADC generation**

Vc-*seco*-DUBA was purchased from MCE (MedChemExpress, Monmouth Junction, New Jersey, US). P5(OEt)-VC-PAB-MMAF was synthesized as previously described. (1) TCEP solution (0.5 M, pH 7.0) and Dulbecco´s PBS was purchased from Merck.

**ADC liquid chromatography/mass spectrometry (LC/MS) analysis**

ADCs were analyzed using a Waters H-class instrument equipped with a quaternary solvent manager, a Waters sample manager-FTN, a Waters PDA detector and a Waters column manager with an Acquity UPLC protein BEH C4 column (300 Å, 1.7 µm, 2.1 mm x 50 mm). Samples were eluted at a column temperature of 80 °C. The following gradient was used: A: 0.1% formic acid in H_2_O; B: 0.1% formic acid in MeCN. 25% B 0-1 min, 0.4 ml/min, 25-95% B 1-3.5 min 0.2 ml/min, 95% B 3.5-4.5 min 0.2 ml/min, 95-25% B 4.5-5 min 0.4 ml/min, 25-95% B 5-5.5 min 0.4 ml/min, 95-25% B 5.5-7.5 min 0.4 ml/min. Mass analysis was conducted with a Waters XEVO G2-XS QTof analyzer. Proteins were ionized in positive ion mode applying a cone voltage of 40 kV. Raw data was analyzed with MaxEnt 1.

**Preparative size-exclusion chromatography**

Protein purification by size-exclusion chromatography was conducted with an ÄKTA Pure FPLC system (GE Healthcare, Chicago, Illinois, US) equipped with a F9-C-fraction collector.

**Sample preparation of ADCs and antibodies for mass spectrometry**

0.5 μl PNGase-F solution (Promega, Madison, Wisconsin, US; recombinant, cloned from Elizabethkingia miricola 10 u/μl) and 5 µl of a 100 mM solution of DTT in water were added to 50 µl of 0.2 mg/ml antibody or ADC in PBS and the solution was incubated at 37 °C for at least 2 h. Samples were subjected to liquid chromatography/mass spectrometry (LC/MS), injecting 2 µl for each sample.

**Analytical size-exclusion chromatography**

Analytical-SEC (A-SEC) of the ADCs was conducted on a Vanquish Flex UHPLC System with a DAD detector, Split Sampler FT (4 °C), Column Compartment H (25 °C) and binary pump F (Thermo Fisher Scientific) using a MAbPac SEC-1 300 Å, 4 x 300 mm column (Thermo Fisher Scientific) with a flow rate of 0.15 ml/min. Separation of different ADC/mAb populations have been achieved during a 30 min isocratic gradient using a phosphate buffer at pH 7 (20 mM Na_2_HPO_4_/NaH_2_PO_4_, 300 mM NaCl, 5% v/v isopropyl alcohol) as a mobile phase. 8 µg ADC/mAb were loaded onto the column for A-SEC analysis. UV chromatograms were recorded at 220 and 280 nm.

**Analytical hydrophobic interaction chromatography**

The measurements were conducted on a Vanquish Flex UHPLC System (2.9) with a MabPac HIC Butyl 4.6 x 100 mm column (Thermo Fischer Scientific). Separation of different ADCs/antibodies have been achieved with the following gradient: A: 1 M (NH_4_)_2_SO_4_, 500 mM NaCl, 100 mM NaH_2_PO_4_ pH 7.4 B: 20 mM NaH_2_PO_4_, 20% (v/v) Isopropyl alcohol, pH 7.4. 0% B: 0-1 min, 0-95% B: 1-15 min, 95% B: 15-20 min, 95-0% B: 20-23 min, 0% B: 23-25 min, with a flow of 700 ul/min. 15 µg sample were loaded onto the column for each analysis. UV chromatograms were recorded at 220 and 280 nm.

**Conjugation of vc-*seco*-DUBA to 20D9h3-mAb to generate 20D9h3-DUBA**

200 μl of the antibody solution at 5.0 mg/ml in PBS were mixed with 2.67 µl of a 10 mM TCEP solution in PBS and the solution was incubated for 1 h at room temperature. Afterwards, 3.33 µl of a 10 mM solution of vc-*seco*-DUBA in dimethyl sulfoxide (DMSO) was added and further incubated for 1 h at room temperature. The reaction mixture was purified by preparative size-exclusion chromatography with a 25 ml Superdex™ 200 Increase 10/300GL (Cytiva) and a flow of 0.8 ml/min eluting with sterile PBS (Merck). The antibody containing fractions were pooled and concentrated by spin-filtration (Amicon® Ultra- 2ml MWCO: 30 kDa, Merck). Details on conjugation and analytics are described in Supplementary Figure 8.

**Conjugation of P5(OEt)-VC-PAB-MMAF to 20D9h3-mAb to generate 20D9h3-MMAF**

50 μl of the antibody solution at 10.0 mg/ml in P5-conjugation buffer (50 mM Tris, 1 mM EDTA, 100 mM NaCl, pH 8.3 at room temperature) were mixed with 3.33 µl of a 10 mM TCEP solution in P5-conjugation buffer. Directly afterwards, 1.67 µl of a 40 mM solution of P5(OEt)-VC-PAB-MMAF dissolved in DMSO were added. The mixture was shaken at 350 rpm and 25 °C for 16 h. The reaction mixtures were purified by preparative SEC with a 25 ml Superdex™ 200 Increase 10/300GL (Cytiva) and a flow of 0.8 ml/min eluting with sterile PBS (Merck). The antibody containing fractions were pooled and concentrated by spin-filtration (Amicon® Ultra- 2ml MWCO: 30 kDa, Merck). Details on conjugation and analytics are described in Supplementary Figure 9.

**Determination of mAb and ADC concentration**

Antibody concentration was determined with Nanodrop. The ADC concentrations were determined in a 96-well plate with a Pierce™ Rapid Gold BCA Protein Assay Kit (Thermo Fisher Scientific) with pre-diluted protein assay standards of bovine gamma globulin (Thermo Fisher Scientific), in accordance with the suppliers’ instructions.

***Ex vivo* mouse serum drug-to-antibody ratio (DAR) analysis**

Mouse serum was freshly drawn and used in the experiments within a couple of hours. 40 μl of normal mouse serum containing the vc-*seco*-DUBA ADC in a concentration of 0.4 mg/ml in at least 80% fresh mouse serum were sterile filtered with UFC30GV0S centrifugal filter units (Merck) and incubated at 37 °C for 6 days. Samples for day 0 were directly processed further. The supernatant of 50 µl anti human IgG (Fc-Specific) agarose slurry (Merck) was removed by centrifugation and the remaining resin washed three times with 300 µl PBS. The resin was incubated with 40 μl of the serum-ADC mix for 1 h at room temperature. Afterwards, the supernatant was removed and the resin washed 3 times with 300 µl PBS. Following by incubation for 5 min with 60 μl 100 mM Glycin buffer pH 2.3 at room temperature. This solution was rebuffered to PBS by using 0.5 ml Zeba™ Spin Desalting Columns with 7K MWCO (Thermo Fisher Scientific). The samples were processed further for MS-measurements, as described above.

**Enzyme-linked immunosorbent assay (ELISA)**

96-well MaxiSorp plates (Thermo Fisher Scientific) were coated with 1 µg/ml human recombinant FLT3 protein (10445-H08H, Sino biological, Bejing, China) or BSA in PBS overnight at 4 °C. On the next day, the plate was treated with 0.5% BSA (w/v) for 1 h at room temperature to block unspecific binding. Afterwards, the plate was incubated with anti-FLT3 antibodies (20D9 and humanized mAbs 20D9h1-20D9h12, in-house) for 2 h followed by incubation with 1:10 000 diluted anti-human alkaline phosphatase coupled rabbit anti-human secondary antibody (309-055-008, Jackson ImmunoResearch, West Grove, Pennsylvania, US) for 1 h in blocking solution, respectively. After each antibody incubation, the plate was washed 5x with PBS, 0.05% Tween 20. Attophos fluorescent substrate (S1000, Promega) was added for 20 min and the plate was read with an excitation of 405 nm and an emission of 500-550 nm on a plate reader (GloMax GM3000).

**Cell culture**

ExpiCHO cells were purchased from Thermo Fisher Scientific, all other cell lines were purchased from DSMZ and cultured as recommended with RPMI, 1% Pen/Strep and 10-20% FCS at 37 °C, 5% CO_2_. Ba/F3 cells stably expressing recombinant surface receptors were generated and cultivated as described (2, 3). All cell lines were authenticated by STR profiling and regularly tested for mycoplasma contamination by qPCR (both Eurofins Genomics, Ebersberg, Germany).

**Immunoblotting**

MOLM-13 cells were treated with 20D9h3-DUBA, 20D9h3-MMAF (both in-house) or PBS for 24 h. Immunoblotting with whole-cell lysates was carried out as described before (4, 5) using the following antibodies: mouse anti-human ATR antibody (sc-515173, Santa Cruz Biotechnology, Dallas, Texas, US), mouse anti-human CHK1 antibody (#2360S, Cell Signaling Technology, Danvers, Massachusetts, US), rabbit anti-human ATR (phosphor Thr1989) antibody (GTX128145, Genetex, Irvine, California, US), rabbit anti-human phospo-CHK1 (Ser345) antibody (#2348, Cell Signaling Technology), mouse anti-human-β-actin (A5441, Merck). For expression analysis of rat FLT3 the following antibody was used: rabbit anti-FLT3 (sc-340, Santa Cruz Biotechnology). Further, we used mouse anti-rabbit IgG-HRP (sc-2357, Santa Cruz Biotechnology) and m-IgGκ BP-HRP (sc-516102, Santa Cruz Biotechnology) as secondary antibodies. Membranes were developed for 2 min using Pierce ECL Plus Western Blotting Substrate (32132, Thermo Fisher Scientific) and subsequently imaged with the Vilber Lourmat Fusion fx7.

**Conjugation with pHrodo Deep Red**

Labelling of anti-human IgG1 secondary antibody (AffiniPure™ Goat Anti-Human IgG, Fcγ fragment specific, 109-005-008, Jackson ImmunoResearch) was performed in-house using the pHrodo Antibody Labeling Kit (Thermo Fisher Scientific) according to the manufacturer’s instructions. In brief, one vial of dye was dissolved in 10 µl of 1 M of bicarbonate solution supplied with the kit. 100 µg of antibody at a concentration of 1.1 mg/ml were mixed with the dissolved dye and incubated for 2 h at room temperature. After incubation, free dye was removed using 0.5 ml Zeba Spin desalting columns (Thermo Fisher Scientific, 7 MWCO). pHrodo is a pH-sensitive dye and only fluorescent upon antibody internalization in the acidic late endosome and lysosome.

**Internalization assay**

Ba/F3 cells stably expressing empty MSCV-IRES-YFP vector (ev) or hFLT3 at low (hFLT3low) or high (hFLT3high) levels were seeded into 48-well plates at a density of 1x10^6^ cells/ml for microscopy or into 96-well plates at a density of 6.25x10^5^ cells/ml for flow cytometry using RPMI1640 medium supplemented with 10% FBS and 10 ng/ml murine IL-3. Unconjugated 20D9h3-mAb (in-house) was complexed with pHrodo-conjugated secondary antibody for 15 min at room temperature. The complex was subsequently added to the cells and incubated at 37 °C. For microscopy, cell samples were taken after 24 h and transferred to an 8-well slide with glass bottom (Ibidi, Gräfelfing, Germany). Spinning disk confocal microscopy was performed on a Nikon TiE microscope equipped with a Yokogawa CSU-W1 spinning disk confocal unit (50 µm pinhole size), an Andor Borealis illumination unit, Andor ALC600 laser beam combiner (405 nm/488 nm/ 561 nm/640 nm), and Andor IXON 888 Ultra EMCCD camera. The microscope was controlled by software from Nikon (NIS Elements, ver. 5.02.00). Images were acquired with a pixel size of 217 nm using a Nikon CFI Apochromat TIRF 60x NA 1.49 oil immersion objective (Nikon). The emission of YFP and the pHrodo Deep Red dye was captured by using a 525/50 nm and a 700/75 nm filter, respectively. For flow cytometry, cell samples were taken at 1 h, 5 h and 24 h, washed twice with PBS, 2% FBS, and analyzed at BD FACSCanto II (BD, Franklin Lakes, New Jersey, US).

**In-Fusion Cloning**

In-Fusion Cloning (In-Fusion® HD Cloning Kit, #102518, Takara Bio, San Jose, California, US) was applied according to manufacturer’s protocol. This ligation-independent and highly efficient cloning technique is based on the annealing of homologous ends of an insert and linearized vector. The linearized vector pCDH-EF1a-eFFly-mCherry, kindly provided by Irmela Jeremias (Addgene plasmid #104833; http://n2t.net/addgene:104833 ; RRID:Addgene_104833 (6), Watertown, Massachusetts, US), was generated using restriction enzymes (EcoR1 and BamH1). The sequence of interest was amplified by specifically designed cloning primers (SnapGene) which contain 15 bp extensions at the 5’ end and 3’ end that were homologous to 15 bp at both ends of the linearized vector: 12.5 μl CloneAmp HiFi PCR Premix (#639298, Takara Bio) 2X, primer fwd (tagagctagcgaattcatggatcttgaaggggaccg, 7.5 μM), primer rev (cagcggccgcggatccctggcgctttgttccagc, 7.5 μM), 10 ng template vector and H_2_O dest. to a total volume of 25 μl. The PCR program was 33 cycles with 98 °C for 10 s, gradient 55 °C for 15 s and 72 °C for 5 s/kb. Successful amplification of DNA was verified using agarose gel, from which the amplicon was then purified using the QIAquick Gel Extraction Kit (#28706 Qiagen, Venlo, Netherlands). For the In-Fusion cloning reaction, 2 μl 5x In-Fusion HD Enzyme Premix, 100 ng of purified PCR insert and 50 ng of linearized vector were adjusted to 10 μl with deionized H_2_O and incubated at 50 °C for 15 min. Stellar Competent Cells were used for transformation. For this, the cells were thawed on ice and 30 μl were moved into a 14 ml round-bottom falcon tube to which 1-2 μl (≈ 5 ng) of the In-Fusion reaction mix was added. After 30 min incubation on ice, cells were heat shocked for 45 s at 42 °C in a water bath and placed on ice for 1-2 min. Following cool down, 500 μl pre-warmed (37 °C) SOC medium was added, and the mix was incubated at 37 °C for 1 h on a shaker at 220 rpm. Then, 100 µl of the suspension were plated on LB-ampicillin (100 ng/ml) agar plates and incubated at 37 °C overnight. Plasmid DNA was then isolated using mini preparation (#27106, QIAprep Spin Miniprep Kit, Qiagen).

**Transient transfection**

This transfection method is based on calcium-chloride precipitation. 7x10^6^ HEK293T cells were seeded in a 10 cm dish. The next day at a confluency of 80-90 %, medium (w/o antibiotics) was changed. After 1 h, 50 µl of 2M CaCl_2_ was added drop-wise to 10 μg endotoxin-free plasmid DNA diluted in 450 μl sterile H_2_O. Then, 500 μl HBS buffer was added drop-wise, mixed by pipetting and incubated for 3-4 min. The cells were then incubated at 37 °C and medium (containing Pen/Strep) was changed after 16-20 h. For further analysis and dependent on purpose, cells were lysed 48 h after transfection.

**Lentivirus production and transduction of Molm-13 cells**

Lentiviruses were produced as previously described (7). 1x10^6^ cells seeded in 1 ml medium were transduced overnight with lentiviral constructs in the presence of 8 µg/ml polybrene (#TR-1003, Sigma, St. Louis, Missouri, US). The next day, cells were washed three times with sterile filtered PBS, and resuspended in RPMI medium containing 10% FBS and 1% Pen/Strep. After three days, marker positive cells were sorted using a FACS Aria III (BD Bioscience). Enriched cells were expanded and re-sorted once to get a marker expression above 96% of cells.

**Flow cytometry analysis of mAb binding, receptor surface expression, apoptosis and cell cycle**

For FLT3-mAb binding studies, Ba/F3-pMIY cells expressing hFLT3, different FLT3 orthologues (cynoFLT3, mFLT3, rFLT3), and paralogues (VEGFR, PDGFRα, CSF-1R, c-KIT), or human FcγR (hFcγRI, hFcγRII and hFcγRIII) were incubated with 1 µg FLT3-mAbs (in-house) or IgG1 control (palivizumab, 404770, AbbVie, North Chicago, Illinois, US) for 1 h at 4 °C. Afterwards, cells were incubated with goat F(ab')2 anti-Human IgG-Alexa Fluor 647 (2042-31, Southern Biotech, Birmingham, Alabama, US) for 30 min at 4 °C and subsequently analyzed with BD FACSCanto II.

To confirm receptor surface expression in transgenic Ba/F3 cells, the following antibodies were used: Alexa-Fluor 647 mouse anti-human CD135 (FLT3, 563494, BD, Franklin Lakes, New Jersey, US), Alexa Fluor 647 mouse anti-human CD309 (VEGFR-2, 560871, BD), Alexa Fluor 647 mouse anti-human CD140a, (PDGFRα, 562798, BD), Alexa Fluor 647 rat anti-human CD115 (CSF-1R, 564945, BD), Alexa Fluor 647 anti-human CD117 (c-KIT, 313235, Biolegend, San Diego, California, US), PE mouse anti-human CD64 (FcγRI, 558592, BD). Cells were stained for 30 min at 4 °C according to the supplier recommendations.

For apoptosis assay, MV4-11 or MOLM-13 cells were treated with 20D9h3-ADCs (in-house) for 24-96 h. Live, dead and apoptotic cells were assessed every 24 h with flow cytometry using APC Annexin V (550475, BD) and DAPI (D9542, Merck). For cell cycle analysis, cells were fixed with 80% ethanol (v/v), stained with propidium iodide (PI), and analyzed by flow cytometry with BD FACSCanto II. The percentage of cells in G1, S and G2/M phase was evaluated with FlowJo version 10.8.1 using the Watson pragmatic algorithm.

**Viability assays**

Cell lines or PDX cells were cultivated with a dilution row of duocarmycin (DC0-NH2, HY-129379, MedChemExpress), exatecan (HY-13631, MedChemExpress), monomethyl auristatin E (MMAE; HY-15162, MedChemExpress), monomethyl auristatin F (MMAF; HY-15579, MedChemExpress), 20D9h3-mAb, 20D9h3-ADCs, IgG1-ADCs (all in-house), gemtuzumab ozogamicin (obtained from Pfizer, New York City, New York, US) or aphidicolin (A0781, Merck) for 24-96 h (as individually stated) at 37 °C, 5% CO_2_. Viable cells were measured either with resazurin assay or trypan blue exclusion as described previously (3), or were counted by flow cytometry using LIVE/DEAD™ Fixable Far-Red Dead Cell Stain or DAPI.

**Bystander assay**

3.3x10^5^ native OCI-AML5 cells/ml were co-cultured with 1.1x10^5^ mCherry-expressing HL-60 cells/ml (ratio of 3:1) and 4.4x10^5^ mCherry-expressing MOLM-13 cells/ml were co-cultured with 5.5x10^4^ native HL-60 cells/ml (ratio of 8:1) in 96-well plates in RPMI1640 with 10% FBS, respectively. The co-cultured cells were treated with a dilution row of 20D9h3-DUBA or 20D9h3-MMAF starting from 12 µg/ml for 5 d at 37 °C, 5% CO_2_. On the day of analysis, they were stained with LIVE/DEAD™ Fixable Aqua Dead Cell Stain Kit (L34966, Thermo Fisher Scientific) for 30 min on ice, washed with PBS and measured at Cytoflex S (Beckmann Coulter, Brea, California, US).

**Real-time reverse transcriptase polymerase chain reaction (RT-PCR)**

For RNA lysates, 1x10^7^ cells were harvested and washed twice with PBS for optimal extraction. RNA was extracted and purified according to the manufacturer’s instructions (RNeasy Mini Kit #374104 Qiagen, Venlo, Netherlands). Following extraction, sample concentrations were measured using NanoDrop (Thermo Fisher Scientific). cDNA synthesis was performed with 1 µg RNA lysate utilizing the cDNA iScript Synthesis Kit (#1708891, Bio-Rad, Hercules, Calilfornia, US). Quantitative real time RT-PCR for ABCB1 was performed on the C1000 Thermal Cycler using iTaq Universal SYBR Green Supermix (#1725121, Bio-Rad). GAPDH was used as reference gene. Samples were quantified in triplicates using the CFX Maestro Software from Bio-Rad. Primers were designed with SnapGene and purchased from Metabion. The following primers were used: ABCB1 Fwd: 5‘-TGA TGT CTC CAA GAT TAA TGA AGG AAT TGG T-3‘; ABCB1 Rev: 5’-CTT TGC CCA GAC AGC AGC T-3’; GAPDH Fwd: 5’- GGA GCG AGA TCC CTC CAA AAT-3’; GAPDH Rev: 5’- GGC TGT TGT CAT ACT TCT CAT GG-3.

**Pharmacokinetic analysis in mouse serum**

20-weeks-old male NSG mice were treated once with 3 mg/kg 20D9h3-MMAF or 20D9h3-DUBA ADCs (n = 1); 24 h, 48 h and 72 h later blood was collected. To determine total ADC within serum, MaxiSorp 96-well plates (Thermo Fisher Scientific) were coated with mouse anti-human IgG Fc specific antibody (#I6260, Sigma) diluted in PBS (required concentration: 1 µg/ml). To determine intact ADC, plates were coated with either 1 µg/ml anti-MMAF (LEV-PAF1-100, Levena, San Diego, California, US) or anti-duocarmycin (clone B6-2-10 from patent WO2016046173A1, produced with human IgG1 backbone in-house using ExpiCHO expression system) anti-payload antibodies. After blocking in PBS + 0.05% Tween + 2% albumin (Thermo Fisher Scientific), 20D9h3-MMAF or 20D9h3-DUBA ADCs were added for standard curves at concentrations ranging from 15.6-100 000 ng/ml or serum samples in required dilutions in blocking solution. Antibodies were detected by HRP-labelled goat anti-human kappa light chain secondary antibody (A18853, Thermo Fisher Scientific) and Ultra-TMB substrate (#34028, Thermo Fisher Scientific). The reaction was stopped using 1 M sulphuric acid (Sigma) and absorption was measured on a plate reader (Tecan Infinite 200 PRO).

**Imaging of colonies**

After CFU assay with AML PDX cells, images of the whole well of the 6-well plate were acquired with Keyence BZ-X810 microscope using PlanApo 2x 0.10/8.50 mm objective (digital zoom: off). 60 to 80 images per well were acquired and stitched together using BZ-X810 Analyzer software. Images of single colonies were acquired with ZEISS Primovert using Axiocam 208 color and Primo Plan-ACHROMAT 4x/0.10 Ph0 objective.

**Mouse work**

Animal handling was performed as described in detail in (7). Mice were randomly distributed in cages with up to five mice each during weaning by animal care takers. Cages were then selected randomly for each group of cells by the experimenter. Within one trial, sex and age of mice was harmonized. No blinding was done, as bioluminescence imaging gives clear measurable values not vulnerable to subjective biases.

Retransplantation trials were performed as „orientation studies“; therefore, five mice per group were included. Engraftment was monitored by repeated bioluminescence imaging (BLI). When mice had at least two BLI images with a total flux above 1x10^8^ photons/s, engraftment was counted as positive. When total flux did not exceed 4x10^6^ photons/s within 100 days after transplantation, engraftment was counted as negative. Mice were sacrificed by cervical dislocation. For AML-388, one mouse within the DUBA-ADC group showed clinical signs of illness 34 d after transplantation; as the mouse had negative imaging signals two days earlier, we assume leukemia-unrelated reasons. The mouse was sacrificed and excluded from analyses.

**Statistical analysis**

Curve-fit, IC_50_ and EC_50_ value calculations and statistical analyses were done with GraphPad Prism 10.1.2. Presented results are shown as mean ± SD of three biological replicates unless stated otherwise. Differences of p < 0.05 were considered as statistically significant. Sample sizes, used tests and multiplicity-adjusted p values are specified in the figure legends. Data was tested for normal distribution and variance homogeneity and the appropriate statistical test was chosen.

# **Supplementary Tables**

**Supplementary Table 1: Evaluation of the humanized FLT3 antibodies**

**Supplementary Table 2: IC_50_ values of ADC payloads in aphidicolin-arrested versus non-arrested cell lines**

**Supplementary Table 3: IC_50_ values of the different ADCs in leukemia cell lines**

**Supplementary Table 4: IC_50_ values of different ADCs in Ba/F3 cell lines expressing FLT3 and/or hFcγRI**

**Supplementary Table 5: AML PDX samples**

**Supplementary Table 6: Colony counts in CFU and LTC-IC assays with AML PDX cells**

**Supplementary Table 7: Characteristics of bone marrow samples from healthy donors**

# **Supplementary Figures**

**
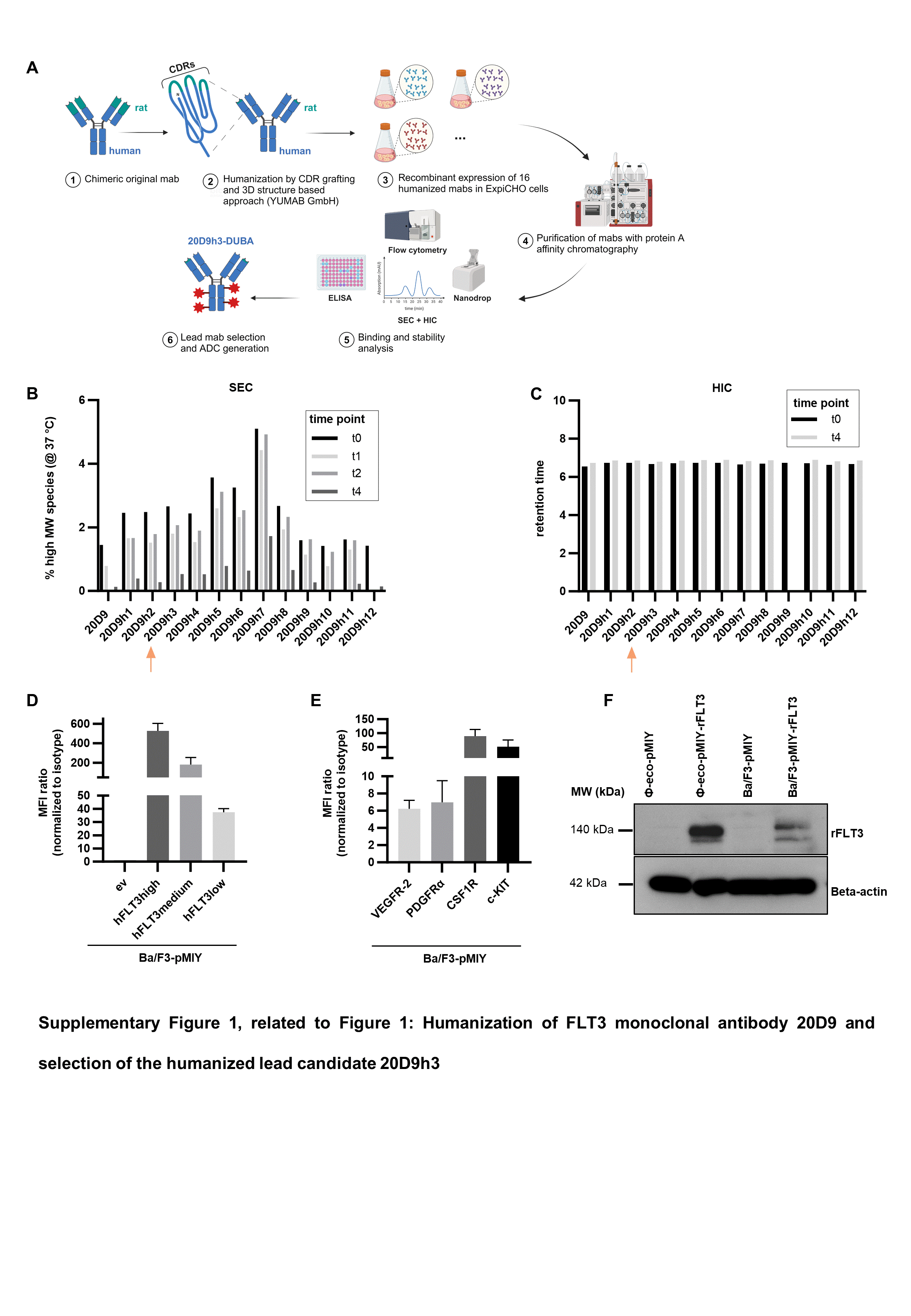
**

**Supplementary Figure 1, related to Figure 1: Humanization of FLT3 monoclonal antibody 20D9 and selection of the humanized lead candidate 20D9h3**

(A) Schematic depiction of 20D9 humanization and production of humanized monoclonal antibodies (mAbs). Humanization was carried out by the company YUMAB GmbH either with CDR grafting or a 3D structure based approach. 16 recombinant mAbs built from four different humanized light and heavy chains were expressed in ExpiCHO cells, whereof four could not be expressed. All mAbs were purified by protein A affinity chromatography. Stability and binding was extensively assessed by flow cytometry, enzyme-linked immunosorbent assay (ELISA), hydrophobicity interaction chromatography (HIC), size-exclusion chromatography (SEC) and Nanodrop measurements. The selected lead candidate 20D9h3 was conjugated with duocarmycin (DUBA) and monomethyl auristatin F (MMAF) to produce ADCs. Image was created with BioRender.com. (B) Aggregate analysis by SEC-HPLC of all 16 humanized antibodies and chimeric rat 20D9 after purification (t0) and after incubation at 37 °C for 1 week (t1), 2 weeks (t2), and 4 weeks (t4). Area under the curve was analyzed for high molecular weight (MW) species and monomeric antibody peaks and percentage of high MW species calculated. (C) Retention times of all 16 humanized antibodies and chimeric rat 20D9 in HIC-HPLC after purification (t0) and after incubation at 37 °C for 4 weeks (t4). (D) Cell surface expression of human wildtype FLT3 in Ba/F3-pMIY ev or hFLT3 cells with three different levels – high (hFLT3high), medium (hFLT3medium) and low (hFLT3low) assessed by flow cytometry. MFI is expressed as ratio to isotype control. Note the break in the y-axis. mean±s.d.; n = 3 biological replicates. (E) Cell surface expression of Ba/F3-pMIY cells expressing FLT3 receptor paralogues VEGFR-2, PDGFRα, CSF1R and c-KIT assessed by flow cytometry. MFI is expressed as ratio to isotype control. Note the break in the y-axis. mean±s.d.; n = 3 biological replicates. (F) Phoenix eco cells transiently and Ba/F3 cells stably expressing pMIY ev or pMIY rat FLT3 (rFLT3). Successful expression was verified by Western blotting using beta-Actin as loading control. MW = molecular weight.

**
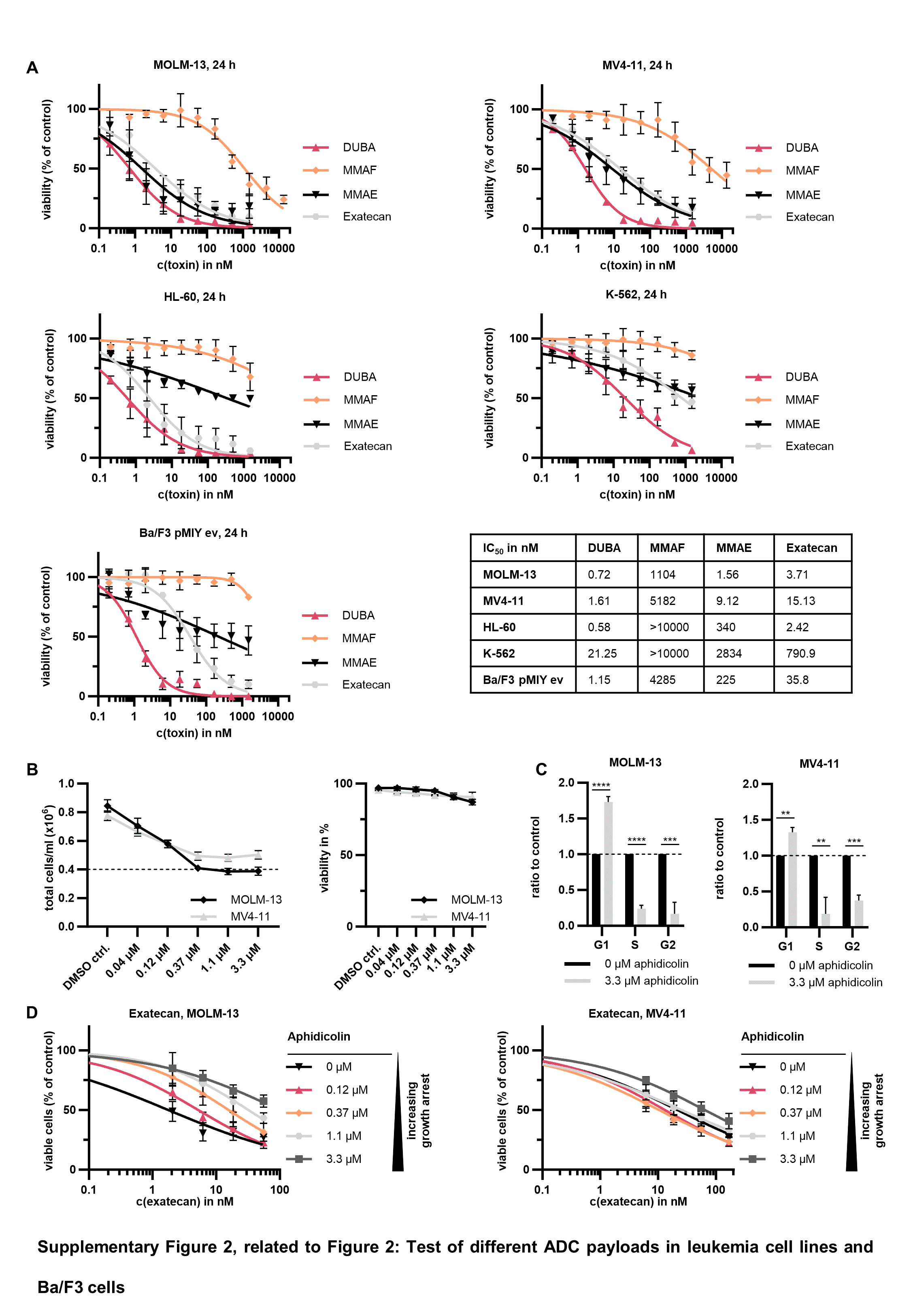
**

**Supplementary Figure 2, related to Figure 2: Test of different ADC payloads in leukemia cell lines and Ba/F3 cells**

(A) MOLM-13, MV4-11, HL-60, K-562 and Ba/F3-pMIY ev cells were treated with a dilution row of MMAF, exatecan, MMAE and DUBA. Viable cells were assessed by resazurin assay or CellTiter-Glo luminescence assay (for Ba/F3 cells) after 24 h and normalized to DMSO-treated control. IC_50_ values were calculated with GraphPad Prism 10.1.2 using nonlinear fit variable slope. mean±s.d.; n = 3 biological replicates. (B) MOLM-13 and MV4-11 cells were treated with 0.04-3.3 µM aphidicolin or DMSO as vehicle-control. After 24 h, ViCell XR was used to assess total cells/ml and viability in % using trypan blue exclusion. Dashed line represents cell density at the time of seeding. mean±s.d.; n = 3 biological replicates. (C) MOLM-13 and MV4-11 cells were treated with 3.3 µM aphidicolin or DMSO vehicle-control for 48 h. Cells were fixed with 80% (v/v) EtOH and stained with PI to evaluate cell cycle phase. Values are depicted as ratio to DMSO control. Dashed line marks untreated control. Unpaired Student´s t test; mean±s.d.; n = 3 biological replicates. (D) MOLM-13 and MV4-11 cells were treated with 0.12 µM-3.3 µM of aphidicolin for 24 h or DMSO vehicle-control. Then, different concentrations of exatecan were added for another 24 h. Viable cells were subsequently assessed with resazurin assay and are depicted normalized to the respective aphidicolin-only control. mean±s.d.; n = 4 biological replicates. **P<.01; ***P<0.001; ****P<.0001; ns, not significant.


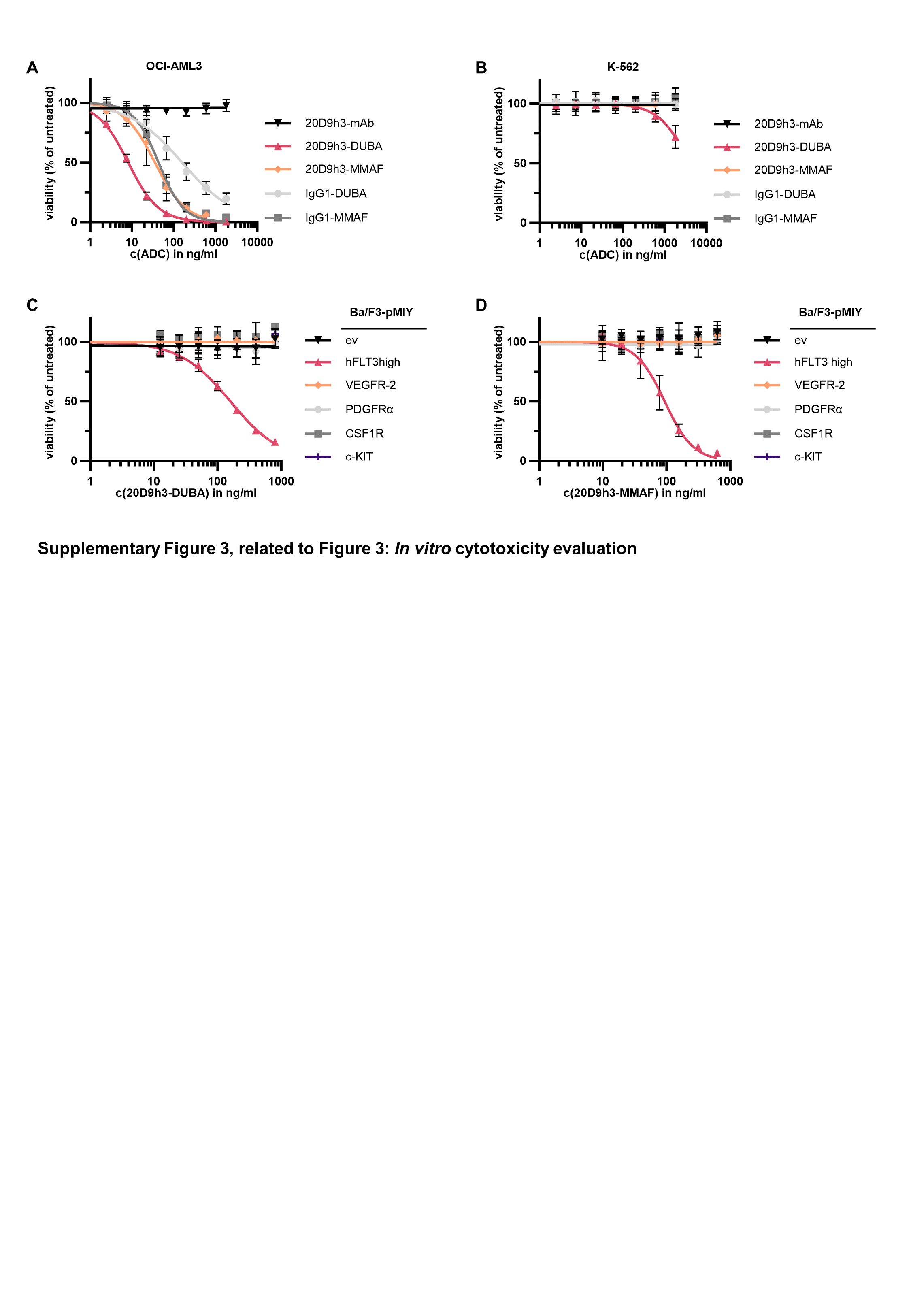


**Supplementary Figure 3, related to Figure 3: *In vitro* cytotoxicity evaluation**

(A) FLT3-positive OCI-AML3 cells and FLT3-negative K-562 cells (B) were treated with different concentrations of 20D9h3-mAb, 20D9h3-DUBA, 20D9h3-MMAF, IgG1-DUBA or IgG1-MMAF antibody-drug conjugates (ADCs) for 96 h. Viable cells were assessed by resazurin fluorescence and normalized to untreated control. mean±s.d; n = 3 biological replicates. (C-D) Treatment of Ba/F3-pMIY ev, hFLT3high, VEGFR-2, PDGFRα, CSF1R and c-KIT cells with a dilution row of 20D9h3-DUBA (C) or 20D9h3-MMAF (D). Viable cells were assessed after 72 h by trypan blue exclusion and normalized to untreated control. mean±s.d.; n = 3 biological replicates.


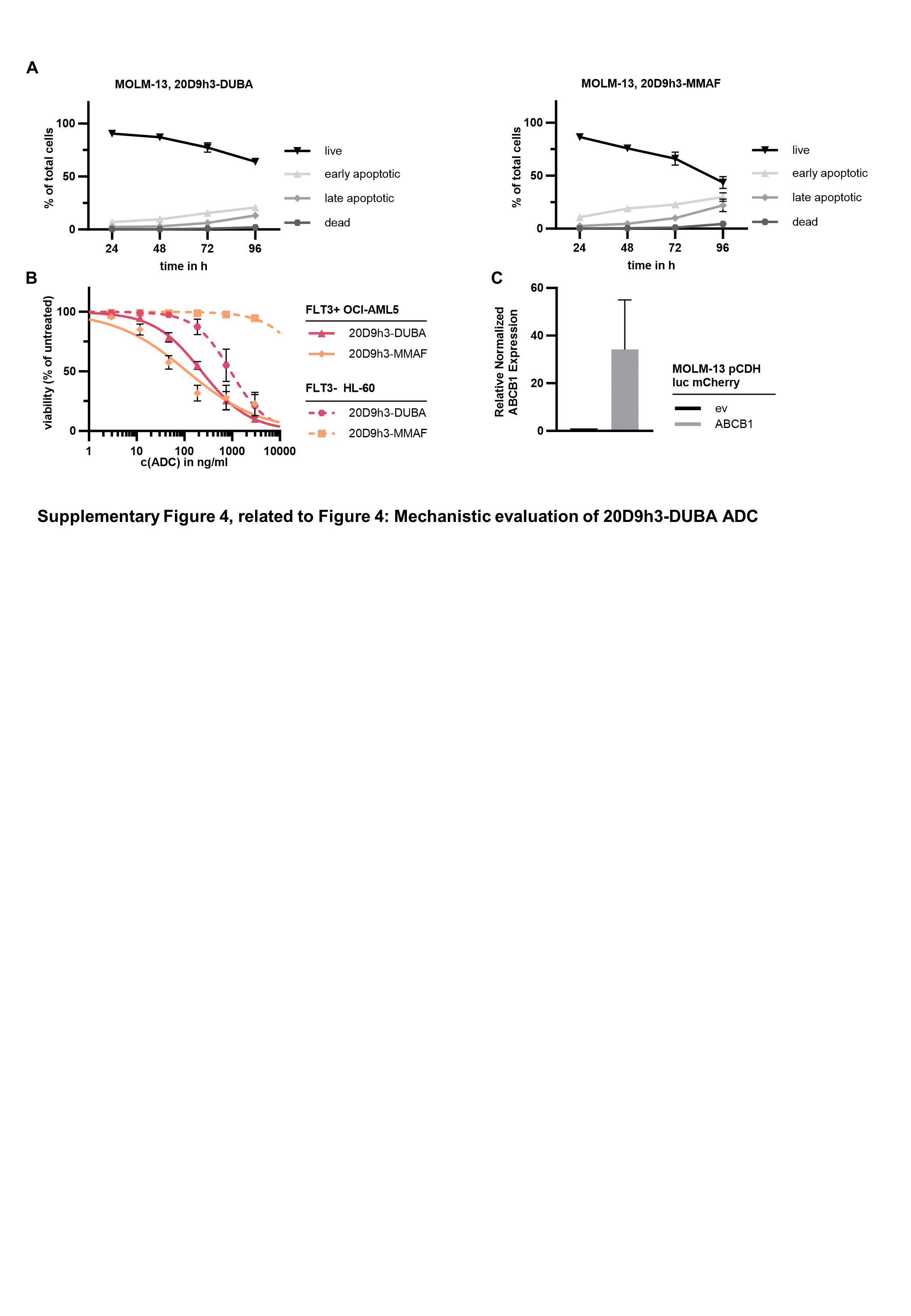


**Supplementary Figure 4, related to Figure 4: Mechanistic evaluation of 20D9h3-DUBA and 20D9h3-MMAF ADCs**

(A) MOLM-13 cells were treated with 100 ng/ml 20D9h3-DUBA (left) or 20D9h3-MMAF (right) for 24-96 h. Every 24 h, viable, apoptotic and dead cells were assessed by flow cytometry using APC Annexin V and DAPI staining. mean±s.d.; n = 4 biological replicates. (B) FLT3-positive native OCI-AML5 cells were co-cultured with FLT3-negative mCherry-expressing HL-60 cells at a ratio of 3:1 for 5 d in the presence of 20D9h3-DUBA or 20D9h3-MMAF. Viable cells were assessed by flow cytometry using LIVE/DEAD™ Fixable Aqua Dead Cell Stain and normalized to untreated control. The two cell types were distinguished by mCherry signal. mean±s.d.; n = 3 biological replicates. (C) Relative normalized expression of ABCB1 assessed by quantitative PCR (qPCR). mean±s.d.; n = 3 biological replicates.


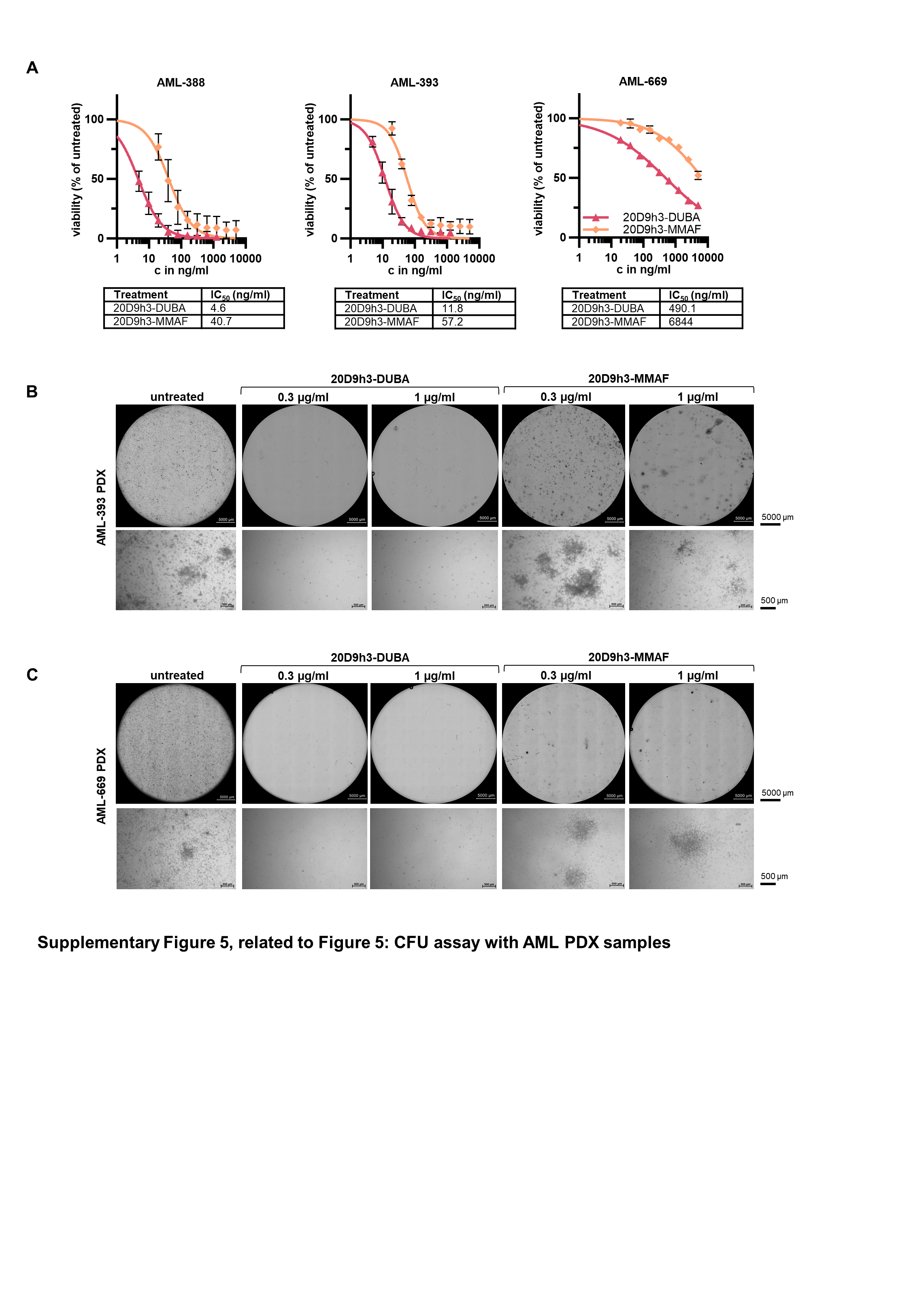


**Supplementary Figure 5, related to Figure 5: CFU assay with AML PDX samples**

(A) AML-388, -393, and -669 patient-derived xenograft (PDX) cells were treated with a dilution row of 20D9h3-DUBA or 20D9h3-MMAF for 4 d. Viable cells were assessed with flow cytometry using DAPI staining and normalized to untreated control. IC_50_ values were calculated with GraphPad Prism 10.1.2 using nonlinear fit variable slope. n = 3 biological replicates. (B, C) Images of AML colony-forming cells (CFCs) for AML-393 (B) and AML-669 (C) on day 14. Upper images show the whole well of a 6-well plate acquired with Keyence BZ-X810 microscope using PlanApo 2x 0.10/8.50 mm objective (digital zoom: off). 60 to 80 images per well were acquired and stitched together using BZ-X810 Analyzer software. Scale bar = 5000 µm. Lower images were acquired with ZEISS Primovert using Axiocam 208 color and Primo Plan-ACHROMAT 4x/0,10 Ph0 objective. Scale bar = 500 µm.


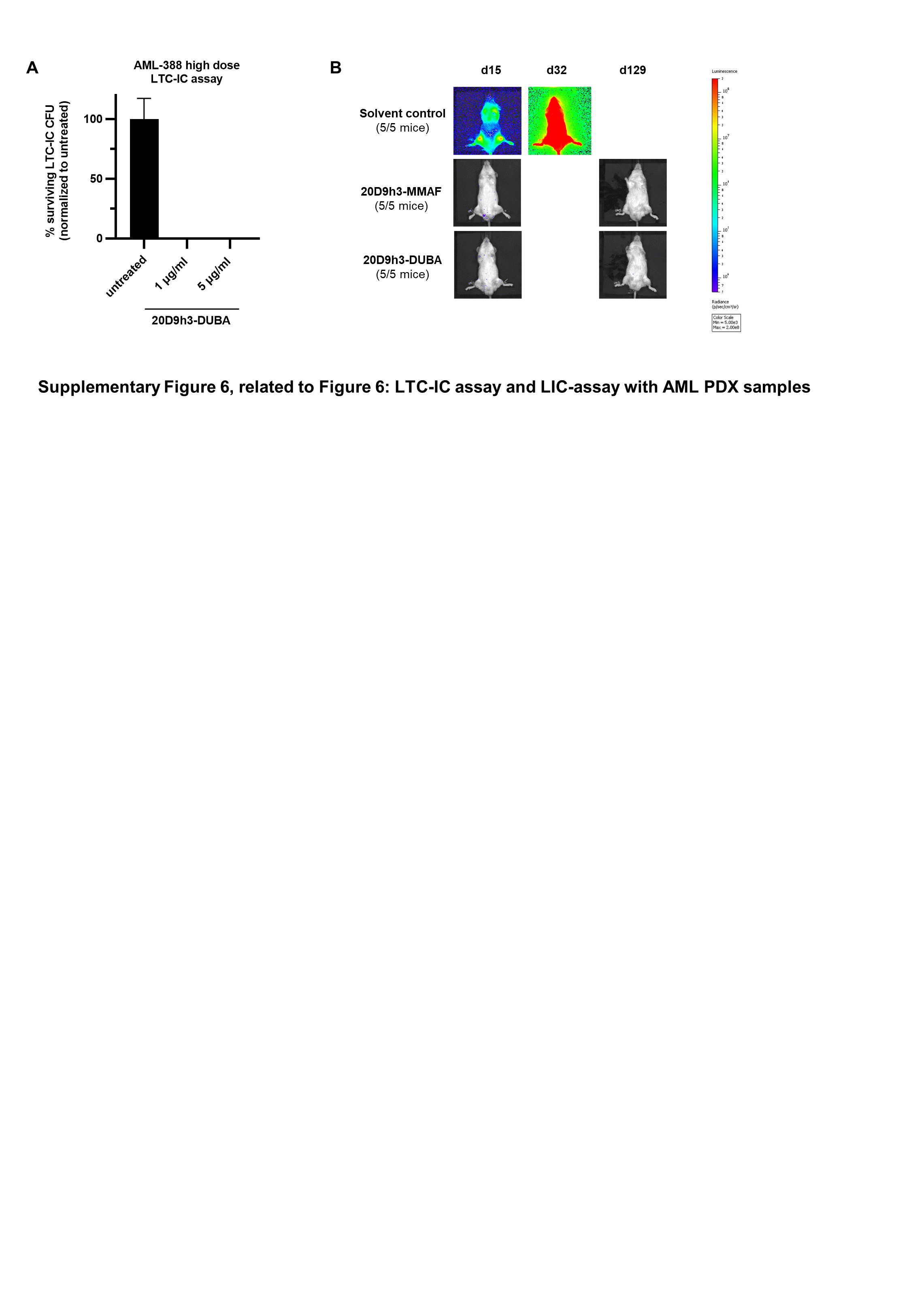


**Supplementary Figure 6, related to Figure 6: LTC-IC assay and LIC-assay with AML PDX samples**

(A) AML-388 PDX cells were treated with high doses (1 and 5 µg/ml) of 20D9h3-DUBA for 4 d and remaining cells were harvested for LTC-IC assay. They were then co-incubated with irradiated SLSL-J-IL3-neo murine fibroblasts in technical duplicates for 5 weeks without further treatment. After 5 weeks, all cells were harvested and plated in methylcellulose in quadruplicates. n = 1 biological replicate including 4 technical replicates. (B) Luc-positive mCherry-positive AML PDX cells of AML-388 (1x10^5^ cells per condition and mouse) were treated *ex vivo* with 1 µg/ml 20D9h3-DUBA or 20D9h3-MMAF for 96 h. On day 4, remaining cells were injected into 21-weeks-old male NSG recipient mice (n=5 per condition) and engraftment was repeatedly monitored by bioluminescence imaging (BLI) for up to 129 d. BLI images of one representative mouse per treatment group are shown. One mouse within the DUBA-ADC group showed clinical signs of illness 34 d after transplantation; as the mouse had negative imaging signals two days earlier, we assume leukemia-unrelated reasons. The mouse was sacrificed and excluded from analyses.


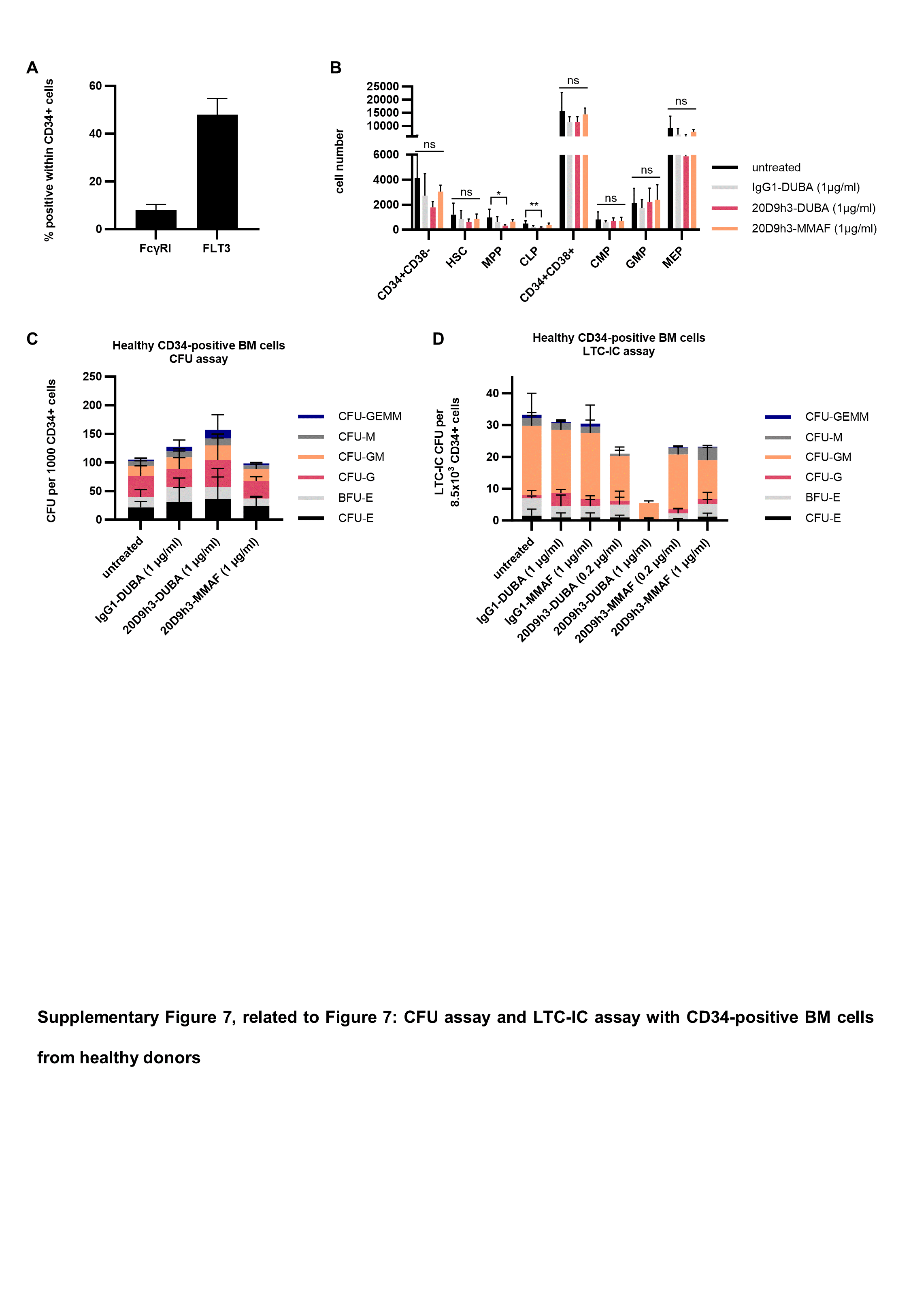


**Supplementary Figure 7, related to Figure 7: CFU assay and LTC-IC assay with CD34-positive BM cells from healthy donors**

(A) FLT3 and FcγRI surface expression on CD34-positive BM cells from healthy donors measured by flow cytometry. mean±s.d.; n = 5 donors. (B) After 4 d ADC treatment cells were assessed by flow cytometry. mean±s.d.; n = 5 donors. *P<.05; **P<.01; ns, not significant by Kruskal-Wallis test. (C) Colony counts after 14 d incubation of ADC pre-treated CD34-positive BM cells in methylcellulose. mean±s.d.; n = 5 donors. (D) Healthy CD34-positive BM cells were treated with 20D9h3-DUBA, 20D9h3-MMAF, IgG1-DUBA or IgG1-MMAF in the indicated concentrations for 48 h. Remaining cells were co-incubated with irradiated M2-10B4 murine fibroblasts for 5 weeks without further treatment. After 5 weeks, all cells were harvested and plated in methylcellulose in technical duplicates. On the final day, colonies were counted. mean±s.d.; n = 2 biological replicates (donor 3 was excluded from analysis due to low colony numbers, which did not allow reliable distinction of the different colony types). CMP: common myeloid progenitors; GMP: granulocyte-monocyte progenitors; MEP: megakaryocyte/erythroid progenitors; CLP: common lymphoid progenitors; MPP: multipotent progenitors; HSC: hematopoietic stem cells. CFU: colony-forming unit; GEMM: granulocyte, erythrocyte, macrophage, megakaryocyte; GM: granulocyte, macrophage; M: macrophage; G: granulocyte; E: erythrocyte; BFU-E: burst-forming unit erythrocyte.

**
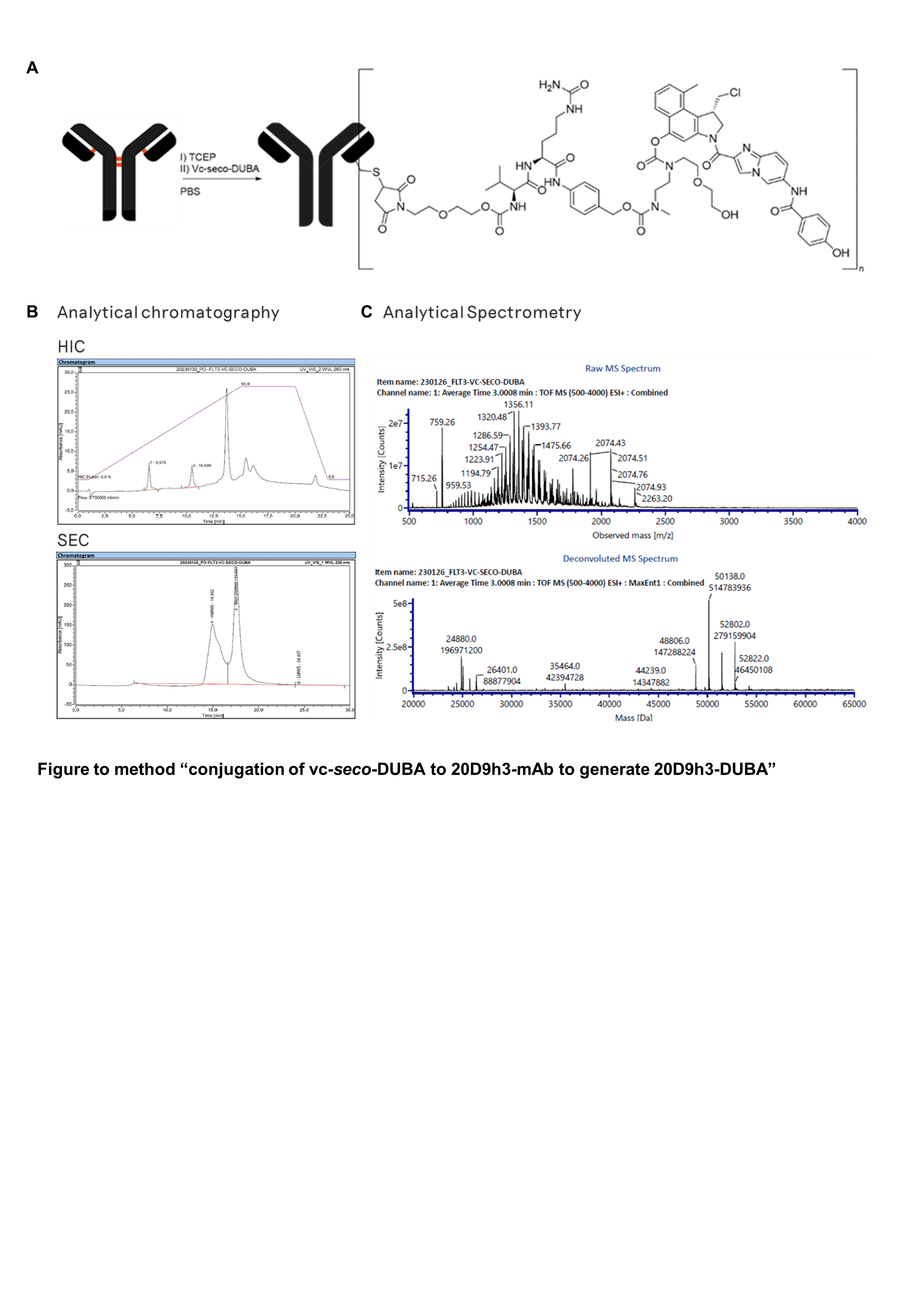
**

**Supplementary Figure 8, related to method “conjugation of vc-*seco*-DUBA to 20D9h3-mAb to generate 20D9h3-DUBA”.** (A) Schematic illustration of 20D9h3-DUBA conjugation. (B) Characterization of 20D9h3-vc-*seco*-DUBA conjugates (20D9h3-DUBA) by hydrophobic interaction chromatography (HIC, top) and size-exclusion chromatography (SEC, bottom). (C) Characterization of 20D9h3-vc-*seco*-DUBA conjugates (20D9h3-DUBA) by mass spectrometry (MS). The crude spectrum is plotted on top and the deconvoluted spectrum on the bottom. Drug-to-antibody ratio (DAR) has been calculated to 4.8 using the intensities of the light and heavy chain species of the deconvoluted spectrum.


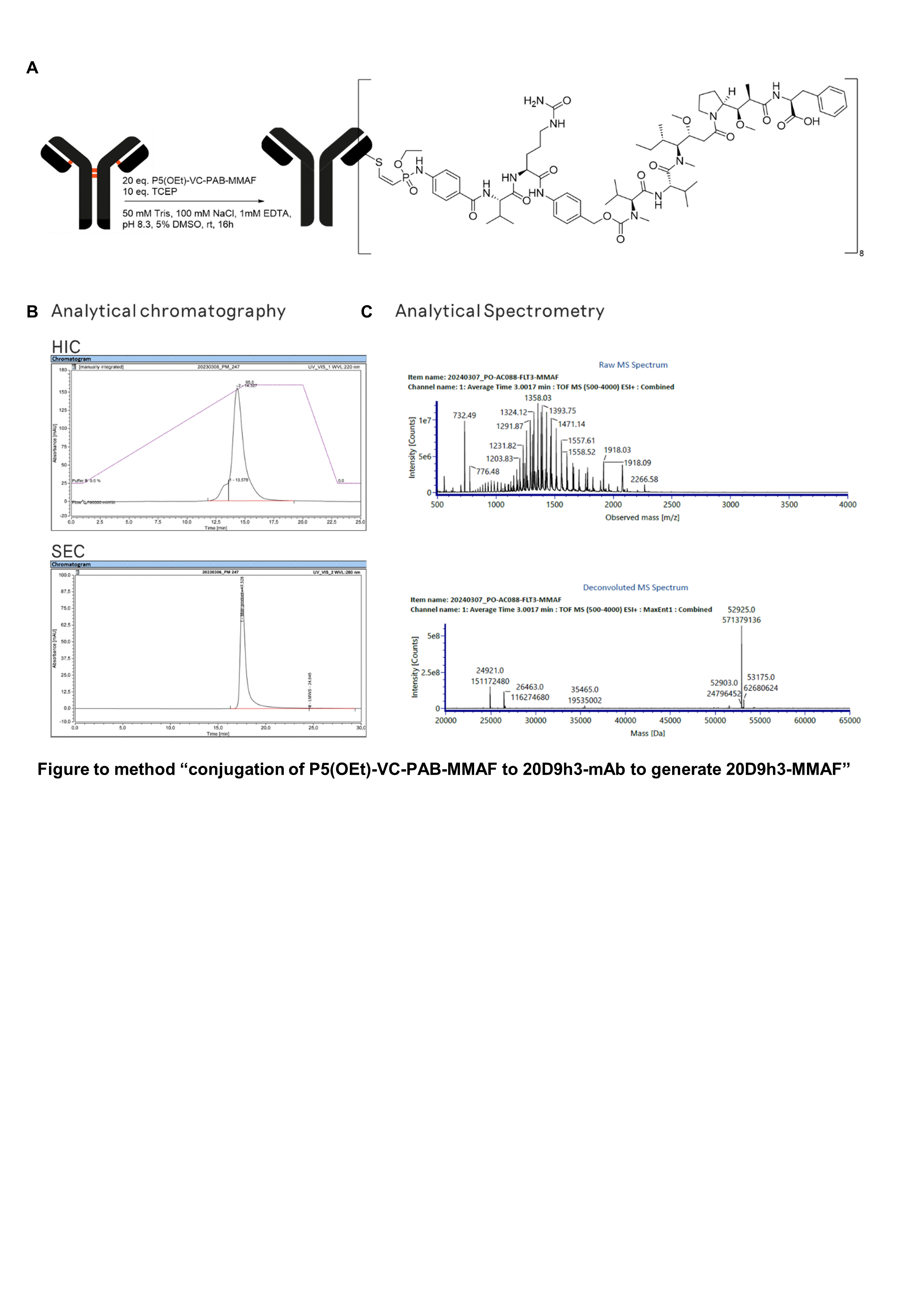


**Supplementary Figure 9, related to method “conjugation of P5(OEt)-VC-PAB-MMAF to 20D9h3-mAb to generate 20D9h3-MMAF”.** (A) Schematic illustration of 20D9h3-MMAF conjugation. (B) Characterization of 20D9h3-P5(OEt)-VC-PAB-MMAF (20D9h3-MMAF) conjugates by hydrophobic interaction chromatography (HIC, top) and size-exclusion chromatography (SEC, bottom) (C) Characterization of 20D9h3-P5(OEt)-VC-PAB-MMAF (20D9h3-MMAF) conjugates by mass spectrometry (MS). The crude spectrum is plotted on top and the deconvoluted spectrum on the bottom. Drug-to-antibody ratio (DAR) has been calculated to 8.0 using the intensities of the light and heavy chain species of the deconvoluted spectrum.


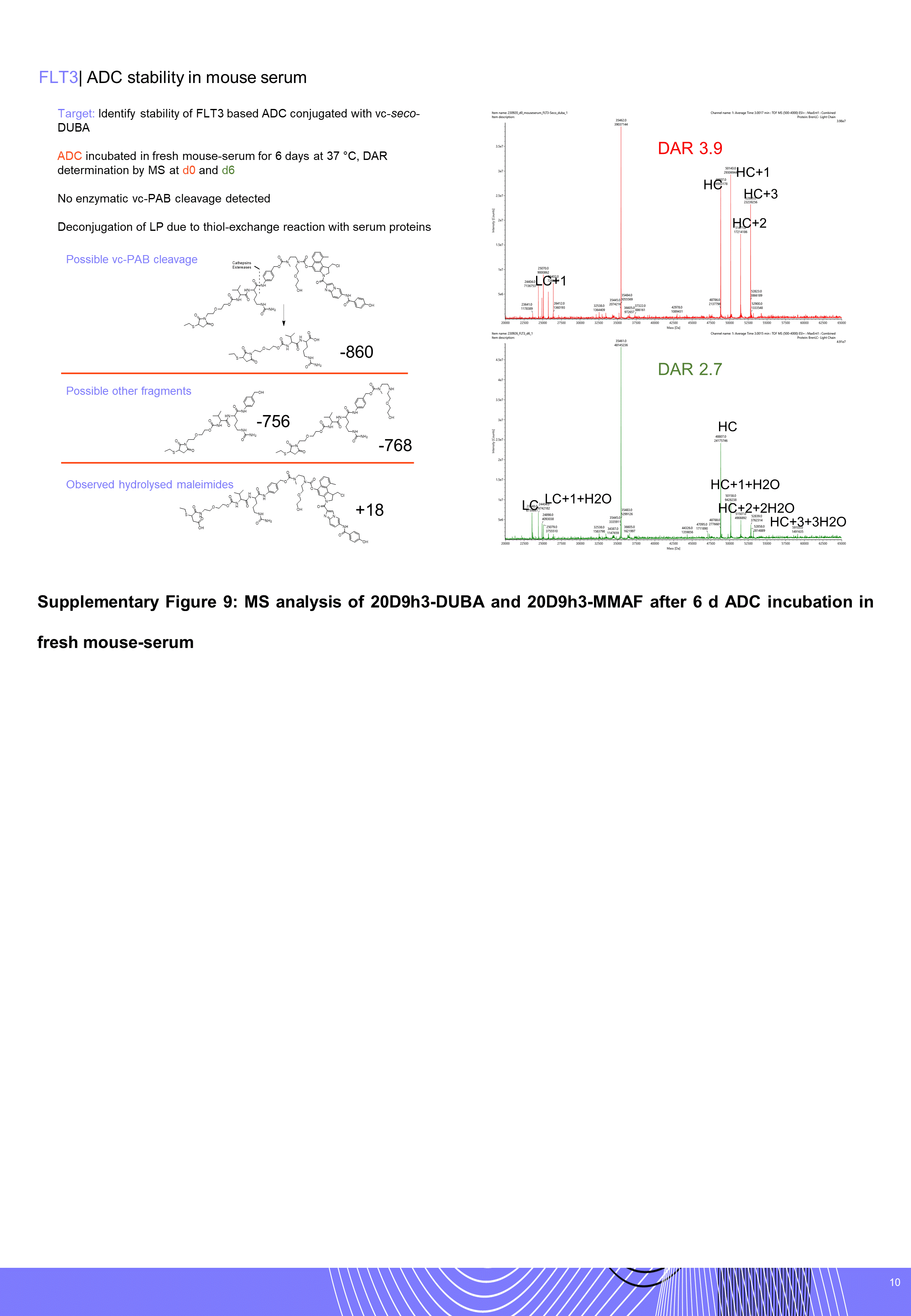


**Supplementary Figure 10: Mass spectrometry analysis of 20D9h3-DUBA and 20D9h3-MMAF after 6 d ADC incubation in fresh mouse-serum.** 20D9h3-DUBA has been incubated with fresh mouse serum at 37 °C for 6 d and analyzed by mass spectrometry (MS) as described above. Potential linker-payload fragments, from CES1-mediated cleavage, drug hydrolysis or maleimide hydrolysis are shown with the indicated masses on the left. MS spectra from day 0 (top) and day 6 (bottom) are shown on the right. Drug-to-antibody ratio (DAR) reduction after 6 days of incubation was observed by linker-payload loss (-1331 Dalton) via retro-Michael addition. The remaining linker-payload that was conjugated appeared as hydrolyzed maleimide (+18 Dalton), as previously described (1). Ces1-mediated cleavage fragments were not observed.


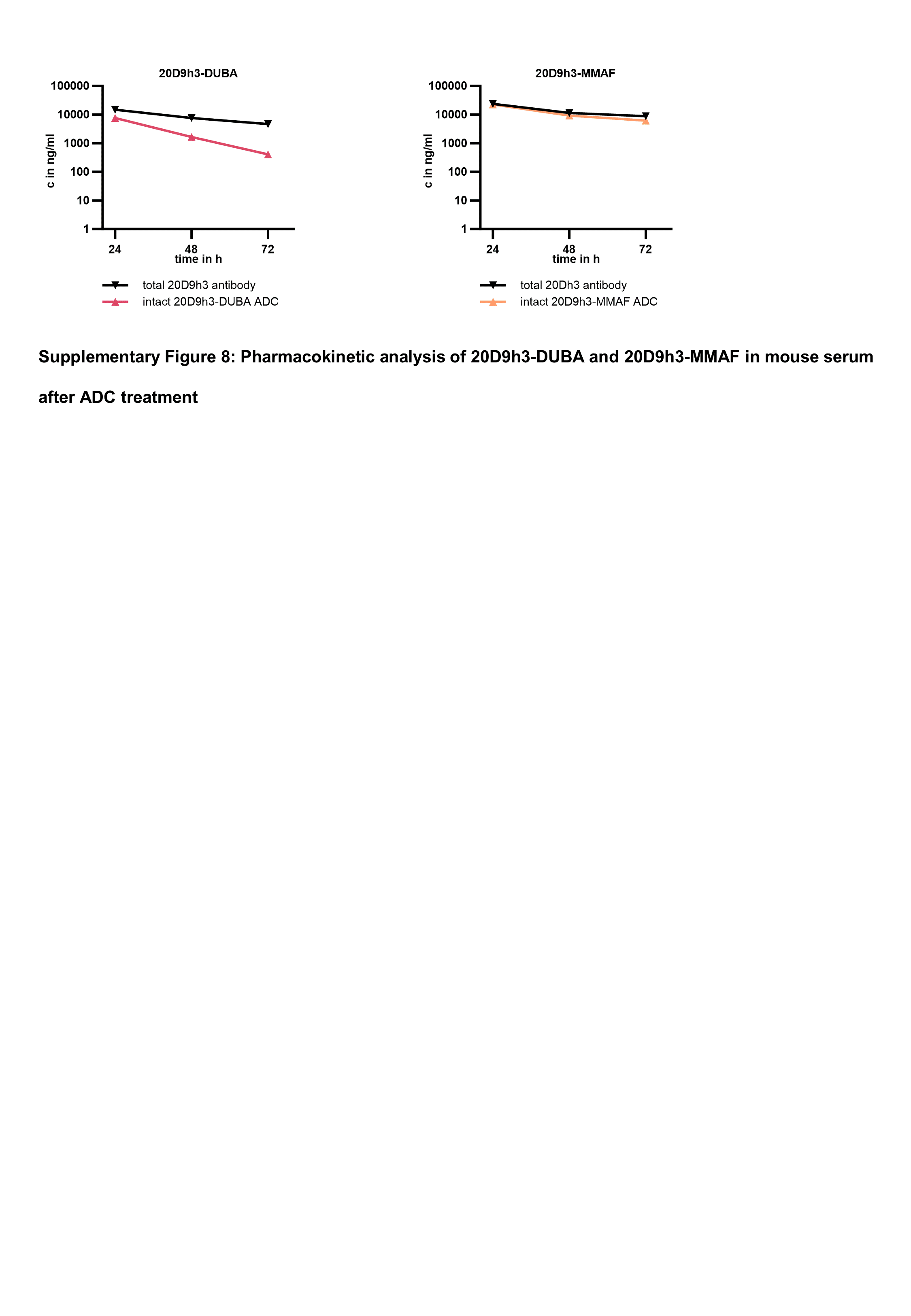


**Supplementary Figure 11: Pharmacokinetic analysis of 20D9h3-DUBA and 20D9h3-MMAF in mouse serum after ADC treatment**

Intact ADC and total antibody were analyzed at different time points in the serum of 20-weeks-old male NSG mice treated once with 3 mg/kg of 20D9h3-MMAF or 20D9h3-DUBA ADC. Total antibody was assessed with anti-human IgG Fc specific antibody, intact ADC was assessed with anti-MMAF or anti-duocarmycin antibody and HRP-labelled goat anti-human kappa light chain secondary antibody in enzyme-linked immunosorbent (ELISA) assay. After addition of Ultra-TMB substrate, plates were measured on Tecan Infinite 200 Pro. mean ± s.d; n = 1 mouse per treatment group with 2 technical replicates per time point.

# **Supplementary References**

1. Kasper MA, Stengl A, Ochtrop P, Gerlach M, Stoschek T, Schumacher D, et al. Ethynylphosphonamidates for the Rapid and Cysteine-Selective Generation of Efficacious Antibody-Drug Conjugates. Angew Chem Int Ed Engl. 2019;58(34):11631-6.

2. Polzer H, Janke H, Schmid D, Hiddemann W, Spiekermann K. Casitas B-lineage lymphoma mutants activate AKT to induce transformation in cooperation with class III receptor tyrosine kinases. Exp Hematol. 2013;41(3):271-80.e4.

3. Roas M, Vick B, Kasper MA, Able M, Polzer H, Gerlach M, et al. Targeting FLT3 with a new-generation antibody-drug conjugate in combination with kinase inhibitors for treatment of AML. Blood. 2023;141(9):1023-35.

4. Stief SM, Hanneforth AL, Weser S, Mattes R, Carlet M, Liu WH, et al. Loss of KDM6A confers drug resistance in acute myeloid leukemia. Leukemia. 2020;34(1):50-62.

5. Kempf JM, Weser S, Bartoschek MD, Metzeler KH, Vick B, Herold T, et al. Loss-of-function mutations in the histone methyltransferase EZH2 promote chemotherapy resistance in AML. Sci Rep. 2021;11(1):5838.

6. Ebinger S, Özdemir EZ, Ziegenhain C, Tiedt S, Castro Alves C, Grunert M, et al. Characterization of Rare, Dormant, and Therapy-Resistant Cells in Acute Lymphoblastic Leukemia. Cancer Cell. 2016;30(6):849-62.

7. Zeller C, Richter D, Jurinovic V, Valtierra-Gutiérrez IA, Jayavelu AK, Mann M, et al. Adverse stem cell clones within a single patient's tumor predict clinical outcome in AML patients. J Hematol Oncol. 2022;15(1):25.
